# Conditioned quantum-assisted deep generative surrogate for particle-calorimeter interactions

**DOI:** 10.1038/s41534-025-01040-x

**Published:** 2025-07-07

**Authors:** J. Quetzalcóatl Toledo-Marín, Sebastian Gonzalez, Hao Jia, Ian Lu, Deniz Sogutlu, Abhishek Abhishek, Colin Gay, Eric Paquet, Roger G. Melko, Geoffrey C. Fox, Maximilian Swiatlowski, Wojciech Fedorko

**Affiliations:** 1 TRIUMF, Vancouver, BC V6T 2A3 Canada; 2 Perimeter Institute for Theoretical Physics, Waterloo, ON N2L 2Y5 Canada; 3 Department of Physics and Astronomy, University of British Columbia, Vancouver, BC V6T 1Z1 Canada; 4 Department of Electrical and Computer Engineering, University of British Columbia, Vancouver, BC V6T 1Z4 Canada; 5 Digital Technologies Research Centre, National Research Council, 1200 Montreal Road, Building M-50, Ottawa, ON K1A 0R6 Canada; 6 Department of Physics and Astronomy, University of Waterloo, Ontario, N2L 3G1 Canada; 7 University of Virginia, Computer Science and Biocomplexity Institute, 994 Research Park Blvd, Charlottesville, VA 22911 USA

**Keywords:** Computer science, Quantum physics, Quantum simulation

## Abstract

Particle collisions at accelerators like the Large Hadron Collider (LHC), recorded by experiments such as ATLAS and CMS, enable precise standard model measurements and searches for new phenomena. Simulating these collisions significantly influences experiment design and analysis but incurs immense computational costs, projected at millions of CPU-years annually during the high luminosity LHC (HL-LHC) phase. Currently, simulating a single event with Geant4 consumes around 1000 CPU seconds, with calorimeter simulations especially demanding. To address this, we propose a conditioned quantum-assisted generative model, integrating a conditioned variational autoencoder (VAE) and a conditioned restricted Boltzmann machine (RBM). Our RBM architecture is tailored for D-Wave’s Pegasus-structured advantage quantum annealer for sampling, leveraging the flux bias for conditioning. This approach combines classical RBMs as universal approximators for discrete distributions with quantum annealing’s speed and scalability. We also introduce an adaptive method for efficiently estimating effective inverse temperature, and validate our framework on Dataset 2 of CaloChallenge.

## Introduction

By the end of the decade, the LHC is expected to begin an upgraded “high luminosity” phase, which will ultimately increase the collision rate by a factor of 10 higher than the initial design. Increasing the number of collisions will generate more experimental data, enabling the observation of rare processes and increased precision in measurements, furthering our understanding of the universe. The path toward the HL-LHC presents great technological challenges and commensurate innovations to overcome them. Monte Carlo simulations of collision events at the ATLAS experiment have played a key role in the design of future experiments and, particularly, in the analysis of current ones. However, these simulations are computationally intensive, projected to reach into millions of CPU-years per year during the HL-LHC run^1^. Simulating a single event with GEANT4^2^ in an LHC experiment requires roughly 1000 CPU seconds. The calorimeter simulation is by far dominating the total simulation time^3^. To address this challenge, deep generative models are being developed to act as particle-calorimeter interaction surrogates, with the potential to reduce the simulation overall time by orders of magnitude. The key point to take into consideration is that one particle impacting a calorimeter can lead to thousands of secondary particles, collectively known as showers, to be traced through the detector, while only the total energy deposit per sensitive element (a cell) is actually measured in the experiment. Hence, through the generation of these showers, non-negligible computational resources are being employed in the detailed recording of the path of these particles. The problem being addressed is whether one can bypass the path-tracing step in the simulation and generate the cell energy deposits directly from a set of well-defined parameters (e.g., type of particle, incident energy, incidence angle, etc.) via sampling from a deep generative model.

There is a large and growing body of literature addressing this critical problem via deep generative models. The earliest methods developed to address this challenge were of the kind of generative adversarial networks^4–6^, which are now an integral part of the simulation pipeline^7,8^ of some experiments. Different deep generative frameworks have been proposed since then, including VAEs^9,9–11^, normalizing flows^12,13^, transformers^14^, diffusion models^15–17^, and combinations thereof^18,19^. A noteworthy development in the field is the CaloChallenge-2022 endeavor^20^, which not only catalyzed research efforts but has also enabled better quantitative comparison across different frameworks. Similarly, defining benchmarks and metrics has been a rather active topic of research^21,22^. A common feature of these models is the fast generation of showers via sampling on GPUs, but further speed increases may still be possible with alternative computing paradigms. In our work, we develop deep generative models which can be naturally encoded onto quantum annealers (QAs), allowing for potentially significant improvements in the speed of shower simulation.

In previous work by this group^23^, a proof of concept of a quantum-assisted deep discrete variational autoencoder calorimeter surrogate called CaloQVAE was presented, whereby the performance in synthetic data generation is similar to its classical counterpart. As a follow-up, in ref. ^24^, we improved our framework by re-engineering the encoder and decoder architectures by introducing two-dimensional convolutions as well as replacing the two-partite graph in the RBM with a four-partite graph, enabling us to use D-wave’s quantum annealer AdvantageSystem^25^. The main contributions of this paper are: (i) we condition the prior on specific incident energies, which allows us to disentangle the latent space embeddings. Most relevant is that we propose a novel method to enforce this condition scheme on the qubits by means of the QA’s flux bias parameters. By adapting flux bias on D-Wave’s systems, we effectively incorporate the flexibility of classical RBM, as universal approximators for discrete distributions, with the potential speedup and scalability of quantum annealing. (ii) We also propose a novel adaptive method to estimate the effective temperature in the quantum annealer. This method is a simple map with a stable fixed point on the effective temperature, and we show it to be more stable with faster convergence than the KL divergence-based method used previously. This contribution is a key methodological advancement that may benefit a wide range of quantum machine learning and sampling applications. (iii) The new model uses 3D convolutional layers for the encoder and decoder as well as periodic boundaries to account for the cylindrical geometry of the shower, leading to an improved performance when compared to its previous counterpart. As the HL-LHC prepares to generate enormous datasets, rapid and accurate simulations are paramount. Our approach addresses an urgent and multidisciplinary challenge, offering a potential solution that may transform simulation pipelines in high-energy physics while contributing to the broader quantum information and machine learning communities. We illustrate our framework by using Dataset 2 of the CaloChallenge. Henceforth, we refer to our framework as Calo4pQVAE.

The paper is organized as follows: In the Results section, we outline the training process of our model and define the criteria used to select the best-performing model. We also showcase the performance of the model using various quantitative and qualitative metrics. In the Discussion section, we interpret the results and provide a thorough discussion on the current limitations of our approach, propose next steps for improvement, and address the technical challenges ahead. In the Methods section, we present the dataset we used, the preprocessing steps employed. This section also introduces the Calo4pQVAE framework, which is detailed in three subsections: the 4p-VAE, where we give a description over the classical framework; the four-partite RBM where we describe in detail the sampling and training procedure for conditioned and non-conditioned RBM; and in quantum annealer we give a brief overview of the motivations behind quantum annealers, explain how they work, and discuss how we incorporate them into our framework, including sampling methods and conditioning techniques.

## Results

In this section, we present the results concerning training and evaluating our model on Dataset 2 of the CaloChallenge. Therefore, we present as results the training aspects of our framework.

### Training

**Fig. 1:**
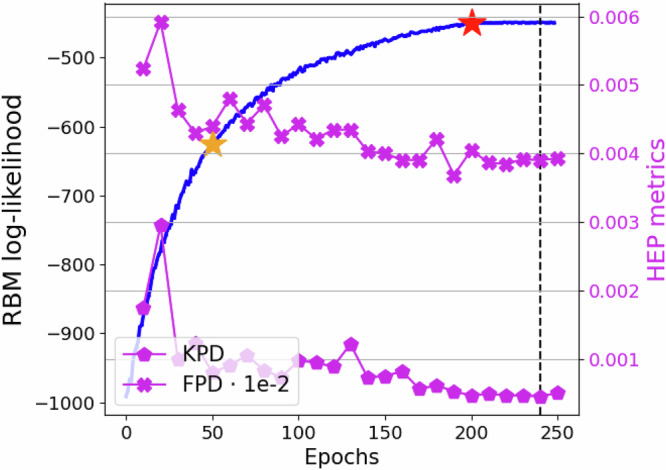
Restricted Boltzmann machine log-likelihood vs epochs. Annealed importance sampling and reverse annealed importance sampling methods were used to estimate the partition function. The annealed step was set to 1/30. The yellow star marks the epoch where the annealed training parameters have reached their final values. The red star marks the point after which the encoder and decoder training parameters are frozen. In purple, we plot the KPD and FPD (right axis) vs epochs.

We train our model for 250 epochs. Each epoch typically has 625 updating steps. The number of block Gibbs sampling steps was set to 3000. During the first 50 epochs, we anneal the model parameters, such as those used in the Gumbel trick, from smooth to a sharp step, to mitigate the discontinuities in the gradient related to the use of discrete variables. After the annealing parameters have reached their final values, we train the model for the next 150 epochs, after which we freeze the encoder and decoder parameters at epoch 200, while the prior distribution parameters keep being updated. We use an Nvidia A100 GPU. Our experiments show that this last step was necessary for the RBM log-likelihood to saturate. In Fig. 1, we show the RBM log-likelihood *vs* epochs. The yellow star points to when the annealed parameters reached their final values, whereas the red star points to when the encoder and decoder parameter were frozen, after which it is clear that the log-likelihood saturates. To estimate the log-likelihood, after training, we used annealed importance sampling and reverse annealed importance sampling methods^26,27^ with an annealed step set to 1/30. As the model updates its parameters, the encoded data used to estimate the log-likelihood will also be modified from epoch to epoch, hence, we stored the validation encoded data for each epoch in order to accurately estimate the RBM log-likelihood. During training, we save an instance of the model every ten epochs.

### Validation

To validate our model, we use Fréchet physics distance (FPD) and the kernel physics distance (KPD)^21^. These are integral probability metrics for high-energy physics, specifically designed to be sensitive to the modeling of shower shape variables. In Fig. 1, we show these metrics *vs* epochs. Henceforth, the results are generated using the 240-epoch model, corresponding to the best KPD metric with a good FPD metric in the saturated RBM log-likelihood regime. We further validate our model’s reconstruction, classical sampling, and QA sampling with GEANT4 data. To sample using the Advantage_system6.4 QA^25^, we first estimate the effective *β* by iteratively using Eq. (24) until the absolute difference between the QA’s energy and that of the RBM is smaller than the reduced standard error, viz.,

1 $$| {\langle H\rangle }_{QA}-{\langle H\rangle }_{RBM}| < \frac{2}{\sqrt{N}}\frac{{\sigma }_{QA}{\sigma }_{RBM}}{{\sigma }_{RBM}+{\sigma }_{QA}}\,.$$

**Fig. 2:**
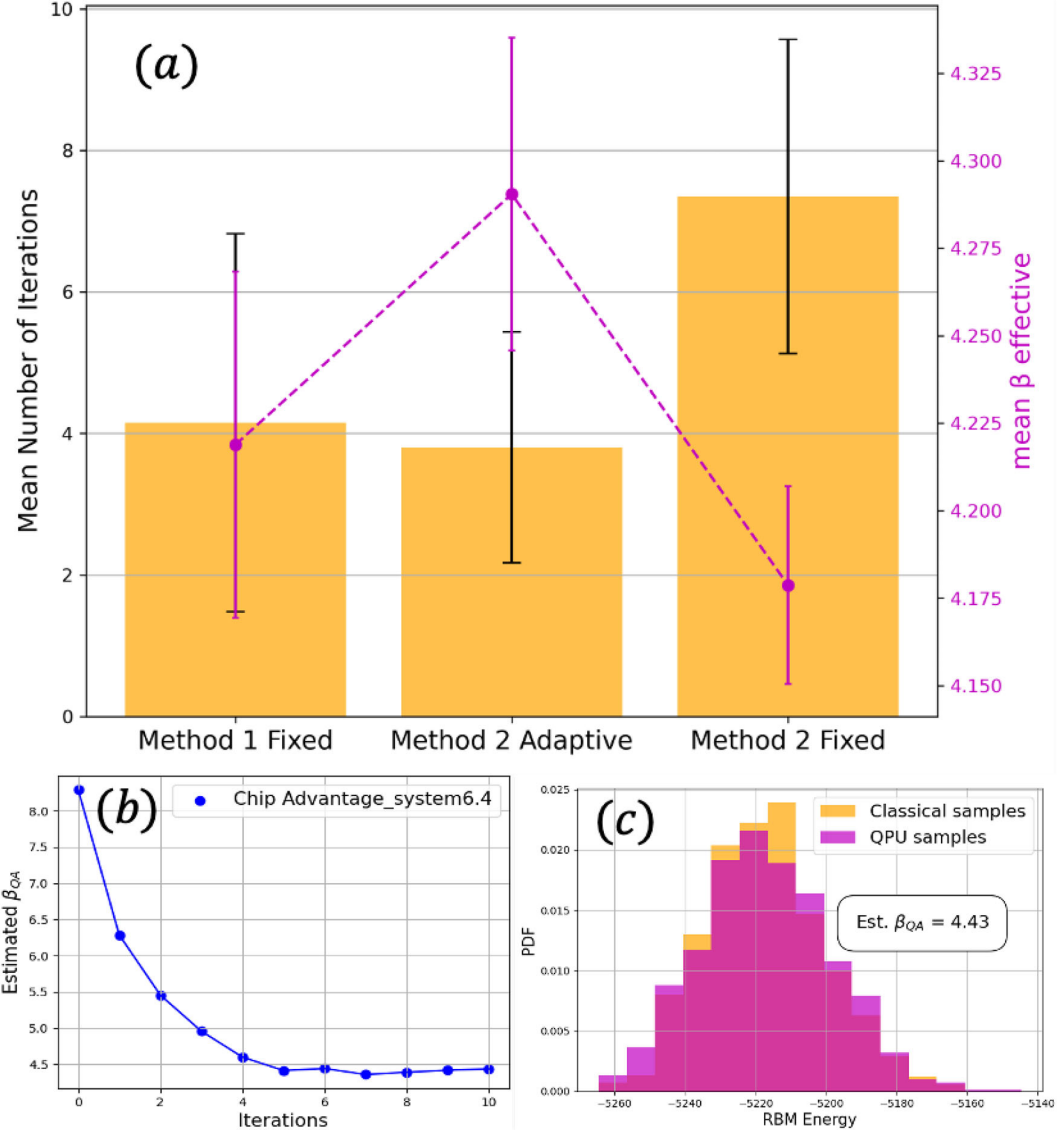
Quantum annealer inverse temperature estimation. **a** Mean number of iterations to meet the reduced standard error threshold in Eq. (1) using different iterative methods. Method 1 uses the KL divergence as in Eq. (23). Method 2 and 3 use Eq. (24), in addition Method 2 adapts the *δ* parameter after each iteration such that *λ* ≈ 0 (see Eq. (25)). The dashed purple line corresponds to the ratio between final minus initial effective *β* and the number of iterations, such that higher values implies faster convergence. The bars correspond to the standard deviation. **b** Estimated inverse temperature vs iterations using method 2, adaptive. **c** RBM histogram using classically generated and QA-generated samples after estimated temperature convergence.

In Fig. 2, we show the mean number of iterations to meet the reduced standard error threshold in Eq. (1) using different iterative methods. Method 1 uses the KL divergence as in Eq. (23). Method 2 uses Eq. (24), where Method 2 adaptive adapts the *δ* parameter after each iteration such that *λ* ≈ 0 from Eq. (25). The dashed purple line corresponds to the ratio between final minus initial effective *β* and the number of iterations, such that higher values implies faster convergence.

In each API call the user specifies the bias and coupler parameters, as well as the number of samples to be generated. The QA is programmed once using these biases and couplers and then performs the annealing process sequentially for the number of iterations requested, returning samples for each anneal. In addition, the user has the option to specify the flux bias parameters with each API call.

**Fig. 3:**
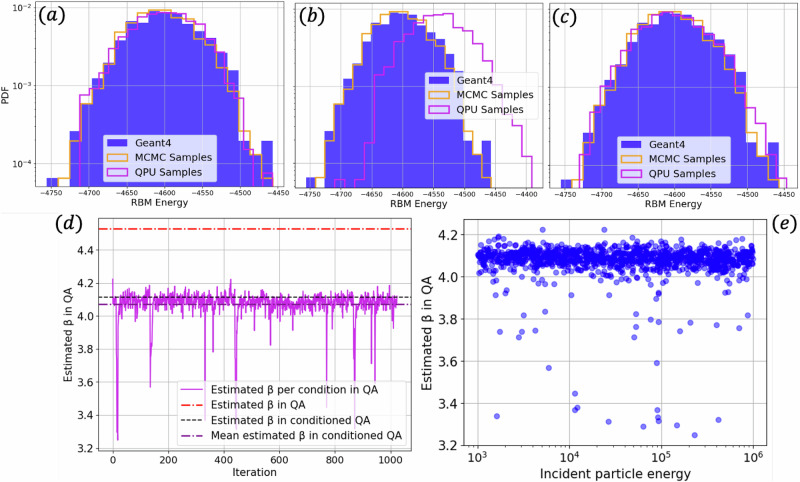
RBM energy histogram obtained from encoded GEANT4 showers, classically generated samples, and QA-generated samples. **a** QA samples obtained via inverse temperature estimation per incident energy condition, **b** single estimated inverse temperature, **c** single estimated inverse temperature and a sleep time of 2.5 s between API call. **d** Estimated inverse temperature per incident energy index in a batch of size 10^4^. The solid purple line corresponds to the obtained inverse temperature for each incident energy condition (the dashed purple line corresponds to the average). The red dashed line corresponds to the estimated inverse temperature in the absence of flux biases. The dashed black curve corresponds to the estimated inverse temperature for the single incident energy condition used to generate the QA samples shown in (**b**, **c**). **e** Estimated inverse temperature vs incident energy condition.

In the case of conditioned sampling using the QA, we estimate the temperature under flux biases. We observed that using flux biases increases the QA’s effective temperature. Additionally, programming the QA increases temperature fluctuations. In Fig. 3a–c, we present the RBM energy histogram obtained from encoded GEANT4 showers, classically generated samples, and QA-generated showers, each panel corresponding to three different experiments we performed. In our first experiment (Fig. 3a), we estimate the inverse temperature for each incident energy condition, as outlined previously, before generating the QA sample. The purple solid line (Fig. 3d) shows the estimated inverse temperature per incident energy condition obtained using Eq. (24) and reaching the threshold in Inequality (1). Notably, we observe rather large but rare fluctuations of the order of 10%. In Fig. 3e, we show the same estimated inverse temperature shown in Fig. 3d versus the incident energy condition, suggesting that these large fluctuations are independent of the incident energy condition. In our second experiment, we repeated the process of generating conditioned samples using the QA, but estimated the QA inverse temperature only once at the beginning of the sampling process. This estimate is depicted as a dashed black line in Fig. 3d. In Fig. 3b, we show the RBM energy, where a small shift in the energy of the QA samples is noticeable. In the third experiment, using the same inverse temperature obtained and used in Experiment 2, we generated new QA samples and introduced a 2.5s pause between QA API calls, that is, per sample. In Fig. 3c, we show the RBM energy obtained from these new samples, from which it is clear that the energy shift has disappeared. Additionally, in Fig. 3d, we show the estimated inverse temperature in the absence of flux biases in dashed red. We conclude that there are two main contributions to the fluctuations: the programming of the QA and the flux biases. The former is responsible for the large but rare spikes in Fig. 3d and can be substantially mitigated by either pausing the sampling process between samples or estimating the inverse temperature per sample. The latter has the effect of shifting the effective inverse temperature to a lower value and can be accounted for by estimating the inverse temperature in the presence of flux biases. We discuss this further in the Discussion section.

**Fig. 4:**
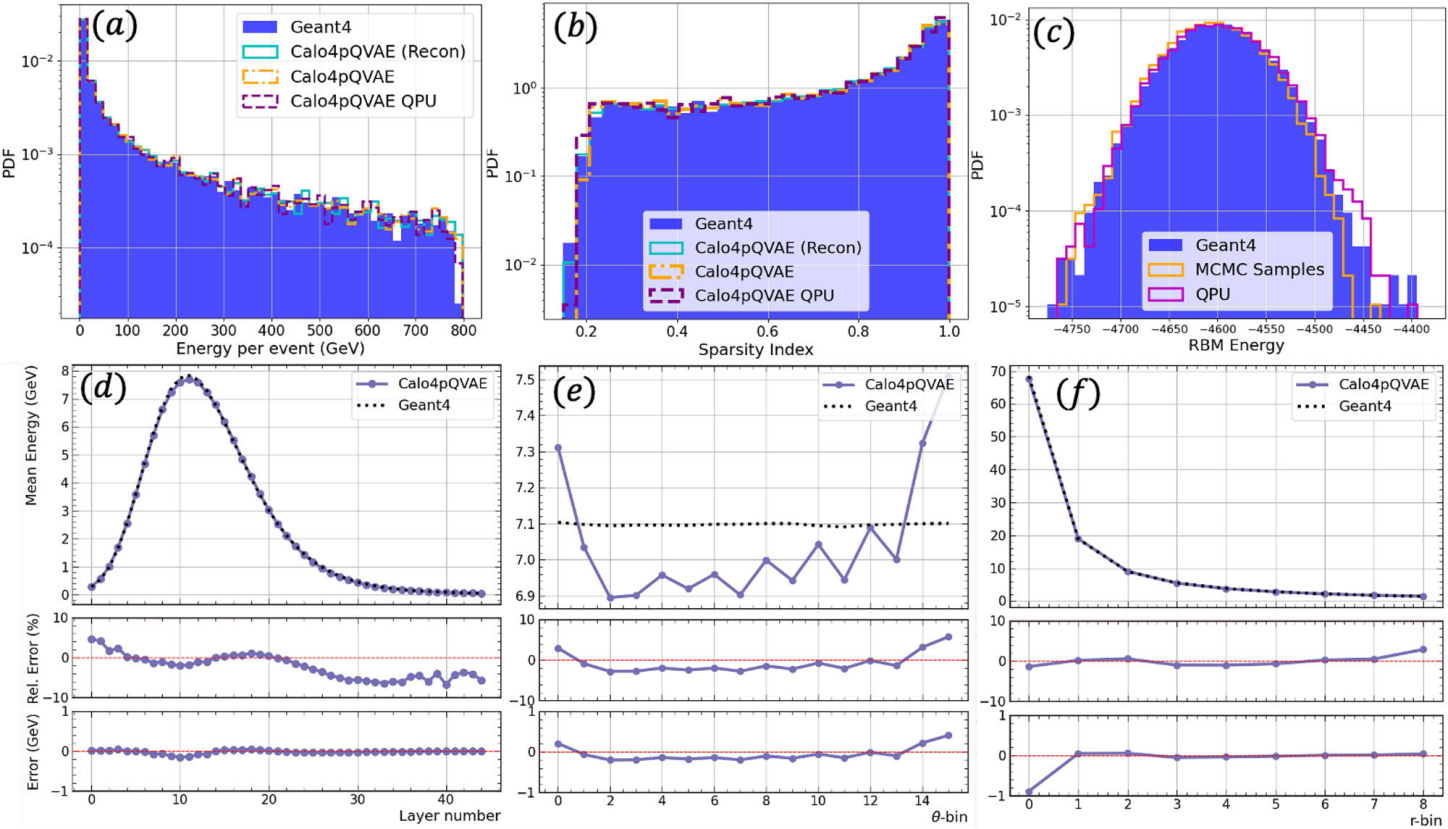
Comparison between GEANT4 samples, reconstructed samples using Calo4pQVAE, classically generated, and QA-generated samples. **a** Energy per event histogram, **b** sparsity index per event, **c** RBM energy per event. Comparison between GEANT4 and classically generated samples in the case of mean event energy vs layer number (**d**), vs angular number (**e**), and vs radial number (**f**). Each panel shows the relative error and the error underneath. The average is taken from 100k events using the Geant4 dataset 2 test data and classically sampled data using Calo4pQVAE.

After estimating the effective *β* in the QA, we use the validation dataset composed of 10,000 data points to benchmark the model. In Fig. 4a, we show the shower energy histograms, while in Fig. 4b, we show the sparsity histogram. The sparsity index is a measure of the sparsity of the shower, and we define it as the ratio between zero-value voxels in the shower and total number of voxels. Each of the histograms include the GEANT4 data and the model’s reconstruction. Furthermore, we test the generative model by generating samples from the RBM and feeding these samples together with the incident energy from the validation dataset to the decoder and generating shower samples. Similarly, we test the quantum-assisted generative model by generating samples from the QA instead of the classical RBM. Both, the classically generated samples and the quantum-assisted generated ones are included in Fig. 4a, b and are labeled sample and sample w/QPU, respectively. In Fig. 4c, we show the RBM energy distribution corresponding to the encoded validation dataset and, both, the classically and QA sampled data.

**Fig. 5:**
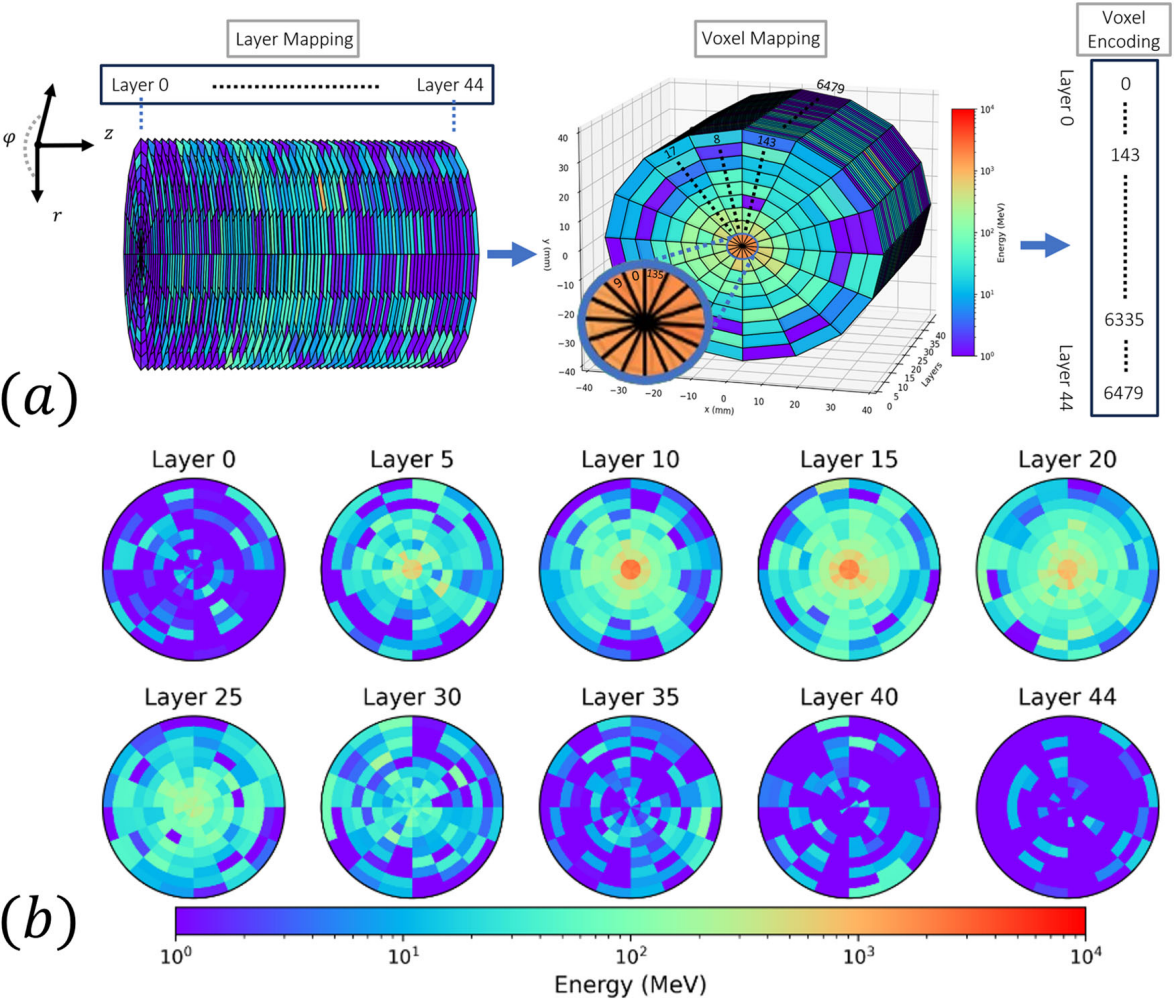
Geometry of Dataset 2 of the CaloChallenge. **a** Calochallenge dataset showers are voxelized using cylindrical coordinates (*r*, *φ*, *z*) such that the showers evolve in the *z* direction. For any given event, each voxel value corresponds to the energy (MeV) in that vicinity. Dataset 2 contains 100k events and the voxelized cylinder has 45 stacked layers. Each layer has 144 voxels composed of 16 angular bins and 9 radial bins. The dataset is parsed onto a 1D vector following the common way to eat a pizza, i.e., grab a slice and start from the inside towards the crust. Each 1D vector has 45 × 9 × 16 = 6480 voxels per each event. **b** Visualization of the voxels in an event in the dataset.

To further validate our model, we classically generate a synthetic dataset composed of 100,000 events with the same incident energy distribution as the training dataset. We compare our synthetic dataset with Dataset 2’s test set^28^ by computing the mean energy in the *r*, *θ,* and *z* directions of the cylinder (see Fig. 5). We show these results in Fig. 4d–f. We observe matching performance with a relative error below 10%. The small overshoot observed at the edges of the mean energy vs the angular variable is likely attributed to the decoder module. As described in the Methods section, we enforce periodic boundary conditions via the encoder. To further refine our approach, we propose imposing periodic boundary conditions directly within the transposed convolutional layers in the decoder in future iterations.

## Discussion

In the previous section, we outlined the process of training our conditioned quantum-assisted Calo4pQVAE and described a novel and faster method to estimate the QA’s effective inverse temperature before sampling using the QPU. We evaluated the performance of our model by means of the KPD and FPD metrics, achieving results on the same order and one order of magnitude higher than CaloDiffusion, respectively, as shown in Table 1^18^. Furthermore, when compared with the models in the CaloChallenge under this metric, our framework performs better than more than half of the 18 models considered. For reference, as a method of relative comparison between models, all models evaluated in the CaloChallenge, except CaloDREAM^14^, yield FPD values at least one order of magnitude above GEANT4, which is of the order of 10^−2^. In the case of KPD, CaloDREAM overlaps with GEANT4 at ~0, while the remaining models produce values ranging from 10^−4^ to 10^−2^ ^20^. Our results demonstrate that (i) our framework is able of reconstructing the showers and preserving sparsity, and (ii) the prior is effectively learning the structure of the encoded data.

**Table 1:** Fréchet particle distance (FPD) and Kernel particle distance (KPD) metrics, implemented in the JetNet^68^ library and adapted for the CaloChallenge^28^

Calo4pQVAE	FPD	KPD
MCMC (100k)	(390.35 ± 1.85) × 10^−3^	(0.46 ± 0.05) × 10^−3^

Another critical metric to evaluate is the shower generation time, which in our framework depends on the decoder processing time and the RBM generation time. Estimating the RBM generation time is not straightforward and there are multiple approaches for this task. This estimation depends on the size of the RBM, its coordination number, and the values of the couplings and biases, among other things. Furthermore, it has been shown that the RBM mixing time increases with training^29^. On the other hand, as mentioned in the Methods section, QA can suffer from freeze-out, depending on features such as the coupling and bias values, the coordination number, and the annealing time. Therefore, benchmarking the classical and quantum-assisted generation times requires careful consideration, and we leave this research for a future paper. Ultimately, both frameworks are several orders of magnitude faster than GEANT4.

We previously mentioned that we set the number of block Gibbs sampling steps to 3000. We observed that with this number of steps, the RBM log-likelihood versus epochs increased monotonically. In this case, generating 1024 samples classically takes ~1 s with ${\mathcal{O}}(1000)$ MCMC steps. Conversely, when sampling with QA, there are three characteristic timescales to consider: the programming time (~10 ms), the annealing time (~20 μ*s*), and the readout time (~100 μ*s*). This results in a total generation time of ~0.1 s for ${\mathcal{O}}(1000)$ samples, i.e., one order of magnitude faster than the classical method, assuming the QPU programming step is performed only once. These estimates per sample are shown in Table 2, which are highly competitive^20^. However, in our current framework, as shown in the Methods section, the flux biases conditioning is done during the programming step, effectively increasing the overall time to ~10 s.

**Table 2:** Shower generation time estimates using GEANT4^15^, Calo4pQVAE on GPUs (assuming 3*k* BGS), and Calo4pQVAE with QPU without conditioning

GEANT4	GPU (A100)	QPU	Anneal time
Time ${\mathcal{O}}(0.1)-{\mathcal{O}}(1{0}^{2})$ s	~2 ms	~0.2 ms	~0.02 ms

Future iterations of D-wave’s QA are expected to decouple the flux biases conditioning from the programming step. Therefore, we anticipate that the quantum-assisted framework to be competitive if the flux biasing conditioning remain below the order of milliseconds. Additionally, uncoupling the flux biases step from the QA programming will help mitigate the undesired large temperature fluctuations. Furthermore, since the self-correlation time is bound to increase with the coordination number, we expect the competitiveness of QAs to improve as the number of couplers per qubit increases.

In the immediate future, we will focus on three key aspects: Exploring RBM configurations: In the present work, we set the number of units in the RBM to 512 units per partition, which implies a compression factor in the Calo4pQVAE of ~30%, similar to that in ref. ^30^. Increasing this number will require increasing the number of block Gibbs sampling steps, as shown in^29,31^. We will explore different RBM sizes with varying numbers of block Gibbs sampling steps to optimize performance and computational efficiency. Enhancing the decoder module: We will investigate the use of hierarchical structures and skip connections in the decoder module, as these can have a positive effect on performance, as observed in diffusion models^18^. Implementing these architectures may improve the model’s ability to reconstruct complex data patterns. Upgrading to the latest quantum annealer: We plan to replace the current QA with D-Wave’s latest version, Advantage2_prototype2.4^32^. Although it has a smaller number of qubits, it offers a greater number of couplers per qubit and reduced noise, which could enhance the quality of quantum simulations and overall model performance. By successfully conditioning a quantum annealer to sample from a desired subspace of data and demonstrating robust, resource-efficient temperature-scaling procedures, our work lays the groundwork for broader applications of quantum annealing in generative modeling. This paves the way for additional quantum training strategies and sets a precedent for feature disentanglement as in ref. ^31^ with quantum hardware.

As a final comment, our framework combines deep generative models with quantum simulations via quantum annealers (QAs). We speculate that the transition to a QA presents new opportunities not only in the noisy intermediate-scale quantum stage but also by paving the way toward utilizing large-scale quantum-coherent simulations^33^ as priors in deep generative models.

## Methods

The CaloChallenge-2022^28^ comprises three distinct datasets, each designed to facilitate research and testing in the field of calorimeter simulations. All three datasets are derived from GEANT4 simulations. The first dataset, referred to as Dataset 1, represents photon and charged pion showers within a specified *η* range (Particle physics experiments typically use a right-hand coordinate system with the interaction point at the center of the detector and the *z*-axis pointing along the beam pipe. The *x*-axis points to the center of the accelerating ring, and the *y*-axis points upwards. Polar coordinates (*r*, *ϕ*) are used to describe the transverse directions of the detector, with *ϕ* being the azimuthal angle around the *z*-axis. The pseudorapidity *η* is defined relative to the polar angle *θ* as η = −ln tan (*θ*/2)). The dataset covers a discrete range of incident energies, ranging from 256 MeV to 4 TeV, logarithmically spaced out evenly with varying sizes at higher energies. The calorimeter geometry is that of the ATLAS detector.

In the case of Dataset 2 it is comprised by two files containing 100,000 GEANT4-simulated electron showers each. These showers encompass a wide energy range, spanning from 1 GeV to 1 TeV sampled from a log-uniform distribution. The detector geometry features a concentric cylinder structure with 45 layers, as shown in Fig. 5, each consisting of active (silicon) and passive (tungsten) material. The dataset is characterized by high granularity, with 45 × 16 × 9 = 6, 480 voxels. The cylinder is 36 radiation lengths deep and spans nine Molière radii in diameter^28^. Lastly, Dataset 3 contains four files, each housing 50,000 GEANT4-simulated electron showers. Similar to Dataset 2, these showers encompass energies ranging from 1 GeV to 1 TeV. The detector geometry remains consistent with Dataset 2 but exhibits significantly higher granularity, boasting 18 radial and 50 angular bins per layer, totaling 45 × 50 × 18 = 40,500 voxels.

These datasets collectively offer a comprehensive resource for researchers interested in the development and evaluation of generative models and simulations within the field of calorimetry in high-energy experiments. The datasets are publicly available and accessible via^34–36^. For our results, we consider Dataset 2 and we leave the testing of our framework using the remaining datasets for future work.

### Data preprocessing

Before feeding the shower and incident energy data to the model, we apply several transformations to the shower and the incident particle energy on-the-fly. Given an event shower, ***v***, and corresponding incident energy, *e*, we first reduce the voxel energy, *v*_*i*_, per event by dividing it by the incident energy, *e*, *viz*.*E*_*i*_ = *v*_*i*_/*e*. Notice that ${E}_{i}\in \left[0,1\right]$, where the left and right bounds correspond to when the voxel energy is zero and equal to the incident energy, respectively. To remove the strict bounds, we define *u*_*i*_ = *δ* + (1 − 2*δ*)*E*_*i*_, where *δ* = 10^−7^, to prevent discontinuities during the logit transformation. Specifically, we use the transformation ${x}_{i}=\ln {u}_{i}/(1-{u}_{i})-\ln \delta /(1-\delta )$, where the second term preserves the zero values in the transformed variable, i.e., when the voxel energy is zero, *v*_*i*_ = 0, the transformed variable *x*_*i*_ = 0.

The incident energy is used as a conditioning parameter and we transform it by applying a logarithmic function followed by scaling it between 0 and 1. These transformations have been used before in the same context^12,15,18^. However, in our case, we modify the process to preserve the zeroes in the transformed variables, *x*_*i*_, and therefore omit the last step of standardizing the new variables, in contrast with other approaches.

### 4p-QVAE

**Fig. 6:**
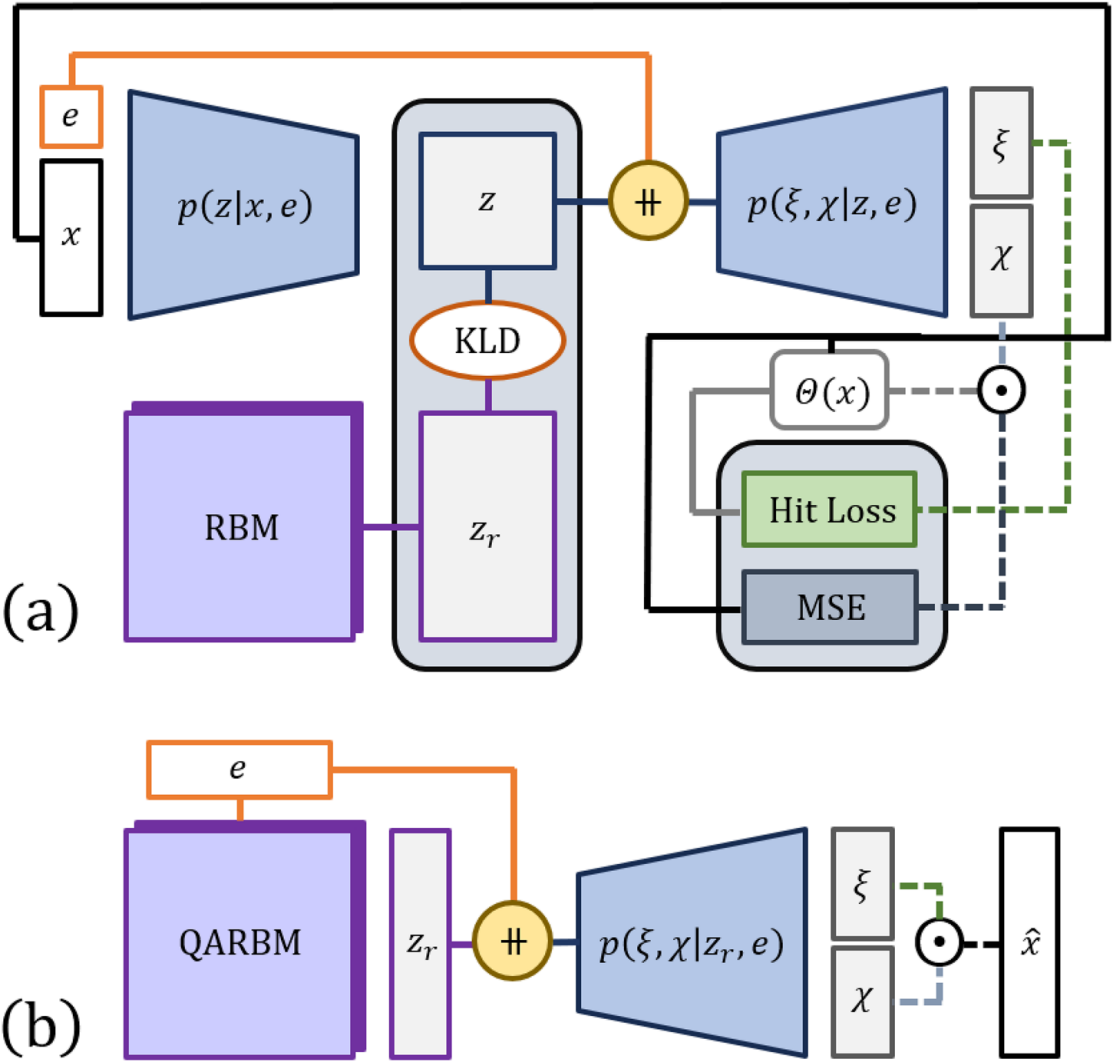
Sketch of Calo4pQVAE. **a** The input data is composed of the energy per voxel, *x* and the incident energy, *e*. During training, the data flows through the encoder and gets encoded into a latent space, *z*, it then goes through the decoder and generates a reconstruction of the voxels per energy, while the incident energy is the label of the event and conditions the encoder and decoder. The decoder outputs the activation vector, *χ* and the hits vector *ξ*. The model is trained via the optimization of the mean squared error between the input shower and the reconstructed shower, the binary cross entropy (hit loss) between the hits vector and the input shower and the Kulbach–Liebler divergence which is composed by the entropy of the encoded sample and the restricted Boltzmann machine log-likelihood. **b** For inference, we sample from the RBM or the QA conditioned to an incident energy, afterwards the sample goes through the decoder to generate a shower.

**Fig. 7:**
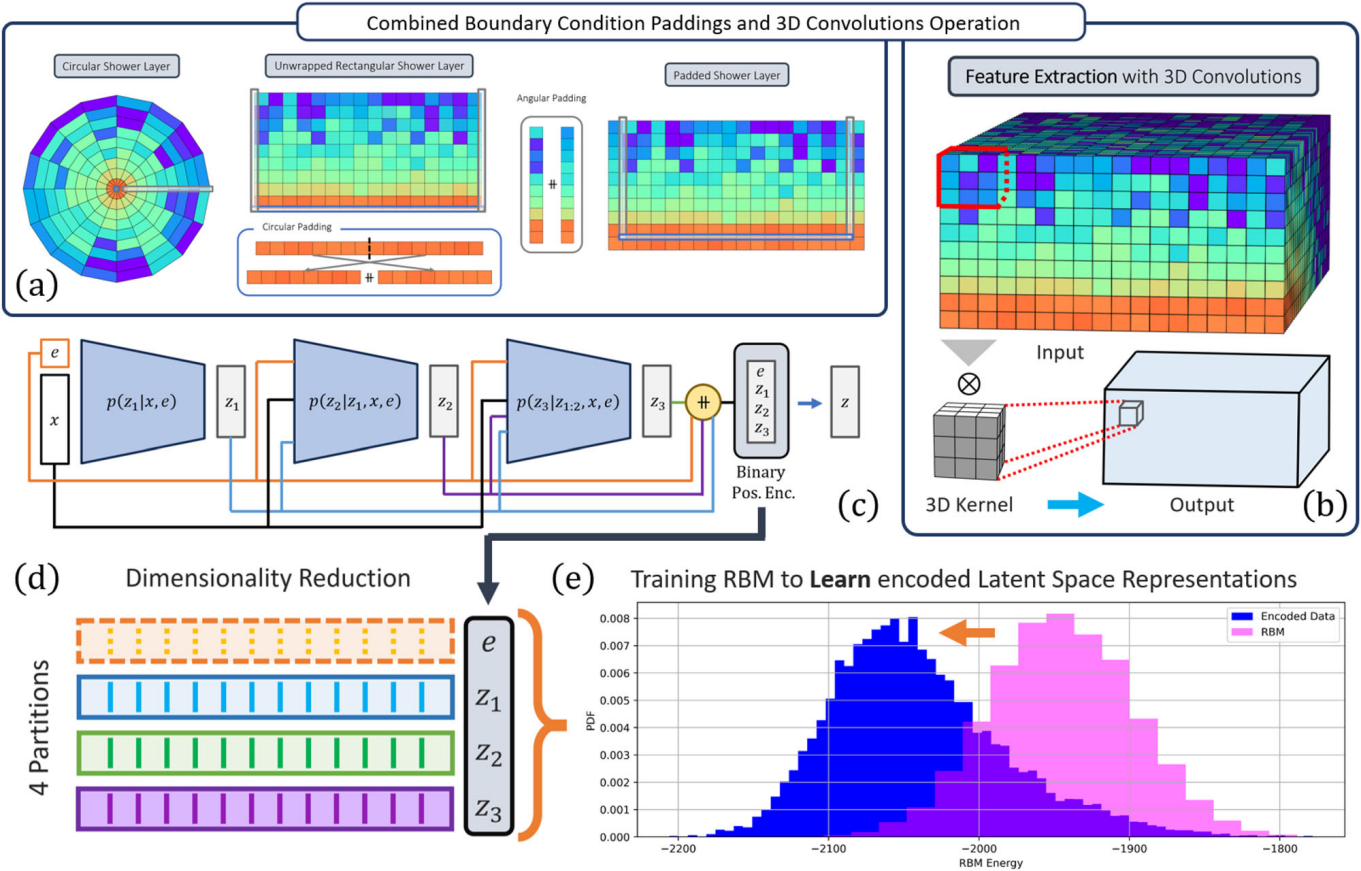
Diagram of the encoding framework. **a** We unwrap the cylindrical shower into a tensor of rank 3 with indices (z,θ,r). We account the angular periodicity of the cylindrical geometry by padding the tensor in θ dimension, such that the size becomes 45 × 18 × 9. To account for the neighboring voxels in the center of the cylinder, we pad the tensor in the corresponding radial dimension. We pad it by taking the centermost voxels, splitting it in half, and permuting the two halves. **b** These operations are performed several times, each prior to a 3D convolution operation for feature extraction. **c** The encoder embeds hierarchy levels, i.e., the first encoder generates a fraction of the encoded data, which is then fed to the second encoder (together with the input) to generate the remaining fraction of the encoded data. The encoded data is used to train the QPU RBM. The encoded data and the incident energy is passed to the decoder to reconstruct the energy per voxel. **d** The Calo4pQVAE uses a discrete binary latent space and assumes a Boltzmann distribution for the prior. The energy function in the Boltzmann distribution corresponds to a sparse 4-partite graph, which allows the direct mapping to the Pegasus-structured advantage quantum annealer. **e** The RBM energy histogram from the encoded showers and the MCMC-generated samples converge during training.

The Calo4pQVAE can be conceptualized as a variational autoencoder (VAE) with a restricted Boltzmann machine (RBM) as its prior, as depicted in Fig. 6. The modularity of our framework allows for the replacement of any component, such as the encoder, decoder, or the RBM, facilitating flexibility in its configuration^30^. The VAE is a widely used framework within deep generative models. Initially, VAEs employed a fixed multivariate Gaussian distribution as their prior^37^. This framework has been studied in the context of the ATLAS electromagnetic calorimeter for photons as a fast shower simulation surrogate^38^. Since its inception, various modifications have been introduced to enhance their capacity to accurately approximate the empirical data distribution^39–42^. In our framework, we use the RBM as the prior, which is known to be a universal approximator of discrete distributions^43^, enhancing the expressivity of the model. The encoder, also referred to as the approximating posterior, is denoted as *q*_*ϕ*_(***z***∣***x***, *e*), while the latent space prior distribution is denoted as *p*_*θ*_(***z***). The decoder, responsible for generating data from the latent variables, is represented as *p*_*θ*_(***x***∣***z***, *e*). We train the model to generate synthetic shower events given a specific incident energy. In other words, we are interested in finding *p*_*θ*_(***x***∣***z***, *e*), such that ∫*p*_*θ*_(***x***∣***z***, *e*)*p*_*θ*_(***z***)*d****z*** matches the empirical dataset distribution. We use *ϕ* to denote the encoder parameters, *θ* for the prior and decoder parameters, and ***z*** the latent space vector. We denote as ***x*** a one-dimensional vector, such that ***x*** ∈ *R*^*n*^, and we say each element *x*_*i*_ contains the energy measured at the *i*th voxel for that specific instance (or event), defined by the bijective transformation described in the previous subsection, while *e* is the incident energy of the event. During training, the model takes as input an instance of ***x*** and *e*. The input data is encoded into the latent space via the encoder. One key difference between VAEs and autoencoders is that in the latter, the same input generally yields the same encoded representation, provided the encoder does not use stochastic filters, such as dropout. In contrast, VAEs generate a different encoded representation with each pass of the same input. This is because the output of a VAE encoder consists of the parameters of a distribution from which the encoded data is sampled. The encoded data is then passed through the decoder, which reconstructs the shower event vector, $\hat{{\boldsymbol{x}}}$. The incident particle energy is the label of the event and conditions the encoder and decoder, as depicted in Fig. 7. To condition the encoder and decoder with the incident energy, we tested two different methods: simple concatenation of the label with the one-dimensional energy per voxel vector, and positional encoding similar to the techniques used by Meta^44^ and Google^45^. Despite experimenting with these different encoding schemes, we observed no significant difference in performance. Therefore, we opted to use the simpler concatenation method henceforth and leave the positional encoding methods for future work when dealing with a greater number of features in the dataset.

The encoder architecture uses hierarchy levels, as described in ref. ^30^. The encoder is composed of three sub-encoders. The first generates one-fourth of the encoded data, which is then fed to the second sub-encoder along with the input data to generate another quarter of the encoded data. This process is repeated with the third sub-encoder, each time integrating all previously generated data and the original input to produce the final encoded output, as depicted in Fig. 7. The purpose of these hierarchy levels is to enforce conditional relationships among latent units by introducing conditioning among latent nodes, viz.

2 $$\begin{array}{lll}{q}_{\phi }({\boldsymbol{z}}| {\boldsymbol{x}})\;\equiv \;{q}_{\phi }({{\boldsymbol{z}}}_{1},{{\boldsymbol{z}}}_{2},{{\boldsymbol{z}}}_{3},{{\boldsymbol{z}}}_{4}| {\boldsymbol{x}})\\\qquad\quad\;\; \,=\;{q}_{\phi }^{(1)}({{\boldsymbol{z}}}_{1}| {\boldsymbol{x}})\mathop{\prod }\limits_{\alpha =2}^{4}{q}_{\phi }^{(\alpha )}({{\boldsymbol{z}}}_{\alpha }| {\{{{\boldsymbol{z}}}_{i}\}}_{i = 1}^{\alpha -1},{\boldsymbol{x}})\end{array}$$

The choice of three sub-encoders is designed on mimicking the connections between the four partitions in the latent space. Our results suggest that this hierarchical approach indeed fosters correlations between latent units, leading to a Boltzmann-like distribution. Additionally, this hierarchical structure introduces multiple paths for gradient backpropagation, similar to residual networks^46^, enhancing the model’s learning capability. The fourth partition conditions the RBM and consists of a binary representation of the incident particle energy, which we describe in detail in Appendix G.

Besides the reconstruction of the event, the encoded data is also used to train the 4-partite RBM via the Kullback–Leibler (KL) divergence. As in traditional VAEs (full derivations can be found in ref. ^37^ and in Appendix A), we optimize the evidence lower bound (ELBO), ${{\mathcal{L}}}_{\phi ,\theta }({\boldsymbol{x}})$, to train the Calo4pQVAE. Explicitly, the ELBO function is:

3 $$\begin{array}{rcl}{{\mathcal{L}}}_{\phi ,\theta }({\boldsymbol{x}})&=&{\langle \ln {p}_{\theta }({\boldsymbol{x}}| {\boldsymbol{z}})\rangle }_{{q}_{\phi }({\boldsymbol{z}}| {\boldsymbol{x}})}-{\left\langle \ln \frac{{q}_{\phi }({\boldsymbol{z}}| {\boldsymbol{x}})}{{p}_{\theta }({\boldsymbol{z}})}\right\rangle }_{{q}_{\phi }({\boldsymbol{z}}| {\boldsymbol{x}})}\,.\end{array}$$

The first term in Eq. (3) represents the reconstruction accuracy and the second term corresponds to the KL divergence. To define a functional form for ${p}_{\theta }(\hat{{\boldsymbol{x}}}| {\boldsymbol{z}})$, certain assumptions about the distribution are typically made. A common assumption is that the likelihood of the reconstruction is Gaussian distributed, which simplifies to optimizing the mean squared error (MSE).

In the case of calorimeter data, one important aspect is differentiating between voxels that are hit (*x*_*i*_ ≠ 0) from those that are not hit (*x*_*j*_ = 0) in a given event. Accurately training deep learning models to produce zero values can be challenging, as typical activation functions for regressions struggle to yield consistent zero outputs. Here we tackle the challenge by separating zero values from non-zero values by factorizing the data vector ***x*** into two components, i.e., ***x*** = ***χ*** ⊙ ***ξ*** where ***χ*** ∈ *R*^*n*^ represents the continuous energy values of the hits and *ξ*_*i*_ = Θ(*x*_*i*_) is a binary vector indicating the presence of hits, with Θ(•) representing the Heaviside function.

Next, we consider a joint probability *p*_*θ*_(***χ***, ***ξ***∣***z***) as the probability of the voxels being hit according to ***ξ*** and energy ***χ***. We can express the joint probability as *p*_*θ*_(***χ***, ***ξ***∣***z***) = *p*_*θ*_(***χ***∣***ξ***, ***z***)*p*_*θ*_(***ξ***∣***z***). We model the event hitting probability, *p*_*θ*_(***ξ***∣***z***), as a Bernoulli distribution $\mathop{\prod }\nolimits_{i = 1}^{n}{p}_{{\xi }_{i}}^{{\xi }_{i}}{(1-{p}_{{\xi }_{i}})}^{1-{\xi }_{i}}$. The number of particles in the electromagnetic shower follows approximately a Poisson distribution. Furthermore, via the saddle point approximation, for a large number of particles in the shower, the multivariate Poisson distribution becomes a multivariate Gaussian distribution with the mean equal to the variance. One can show that the variables ${\{{\chi }_{i}\}}_{i = 1}^{n}$ are also approximately Gaussian distributed, provided *v*_*i*_/*e* ≪ 1 (see App. H). In the present paper, we assume a variance equal to unity in our generative model, as we observed better performance for this choice. Hence, the joint distribution *p*(***χ***, ***ξ***∣***z***) can be formally expressed as

4 $$\begin{array}{ll}{p}_{\theta }({\boldsymbol{\chi }},{\boldsymbol{\xi }}| {\boldsymbol{z}})=\mathop{\prod }\limits_{i=1}^{n}\left({\xi }_{i}\frac{1}{\sqrt{2\pi }}{e}^{-\frac{{({\chi }_{i}-{x}_{i})}^{2}}{2}}\right.\\ \qquad\qquad\qquad+\,\left.(1-{\xi }_{i})\delta ({\chi }_{i})\right){p}_{{\xi }_{i}}^{{\xi }_{i}}{(1-{p}_{{\xi }_{i}})}^{1-{\xi }_{i}}\,.\end{array}$$

Maximizing the log-likelihood of *p*_*θ*_(***χ***, ***ξ***∣***z***) is rather difficult due to the factor containing the Dirac delta function. It may well be possible to replace the Dirac delta function with a smooth parameterized function. Yet, there is a simpler way where we instead mask ***χ*** with ***ξ***, in which case we can neglect the term containing the Dirac delta function. The key point to stress here is that by means of the mask, we split the tasks between generating zeroes and non-zeroes, and generating the voxel energy via ***ξ*** and ***χ***, respectively. Therefore, the joint distribution in Eq. (4) becomes (We removed the *ξ* coefficient due to normalization.):

5 $$\begin{array}{rcl}{p}_{\theta }({\boldsymbol{\chi }},{\boldsymbol{\xi }}| {\boldsymbol{z}})&=&\mathop{\prod }\limits_{i=1}^{n}\frac{1}{\sqrt{2\pi }}{e}^{-\frac{{({\chi }_{i}\cdot {\xi }_{i}-{x}_{i})}^{2}}{2}}\\ &&{p}_{{\xi }_{i}}^{{\xi }_{i}}{(1-{p}_{{\xi }_{i}})}^{1-{\xi }_{i}}\,.\end{array}$$

The reconstruction term transforms to

6 $$\begin{array}{rcl}{\left\langle \ln {p}_{\theta }({\boldsymbol{\chi }},{\boldsymbol{\xi }}| {\boldsymbol{z}})\right\rangle }_{{q}_{\phi }({\boldsymbol{z}}| {\boldsymbol{x}})}=\left\langle \mathop{\sum }\limits_{i=1}^{n}\left[-{\left({\chi }_{i}\cdot {\xi }_{i}-{x}_{i}\right)}^{2}\right.\right.\\ {\left.\left.+{\xi }_{i}\ln ({p}_{{\xi }_{i}})+(1-{\xi }_{i})\ln (1-{p}_{{\xi }_{i}})+\text{const}\right]\right\rangle }_{{q}_{\phi }({\boldsymbol{z}}| {\boldsymbol{x}})}.\end{array}$$

While Eq. (5) outlines the functional structure of the reconstruction of our generative model, the formulation of the loss function requires further consideration. Specifically, there are three key aspects to address. First, the Bernoulli-distributed hits vector is designed to capture the hits distribution in the dataset, yet binary entropy alone does not adequately represent this. Next, the MSE term in Eq. (6) is biased towards low energy when the hits vector reconstruction, ***ξ***, incorrectly predicts values during training. To tackle both concerns, we replace *ξ* → *Θ*(*x*_*i*_), which converts the binary entropy into a binary cross entropy (BCE). The third consideration involves how BCE penalizes errors. In its standard form, BCE penalizes all incorrect hit predictions equally. However, it is intuitive that this should not be the case. For instance, predicting a voxel hit of 0 when the true value is 100 GeV should be penalized more heavily than predicting 0 when the true value is 1 GeV. To account for this, we experimented with reweighting the BCE terms proportional to the voxel value. Despite this modification, we did not observe a significant improvement.

Maximizing the ELBO function leads to maximizing the log-likelihood of *p*_*θ*_(*x*). In practice, VAEs are trained by minimizing the negative ELBO function, from which it is straightforward to notice that the first term in the r.h.s. of Eq. (6) becomes mean squared error (MSE), while the second and third terms correspond to the binary cross entropy. After taking these considerations into account, flipping the sign in the ELBO function and neglecting constant terms, Eq. (6) becomes:

7 $$\begin{array}{c}-{\left\langle \ln {p}_{\theta }({\boldsymbol{\chi }},{\boldsymbol{\xi }}| {\boldsymbol{z}})\right\rangle }_{{q}_{\phi }({\boldsymbol{z}}| {\boldsymbol{x}})}={\left\langle \mathop{\sum }\limits_{i = 1}^{n}{\left({\chi }_{i}(\theta )\cdot \Theta ({x}_{i})-{x}_{i}\right)}^{2}\right\rangle }_{{q}_{\phi }({\boldsymbol{z}}| {\boldsymbol{x}})}\\ -{\left\langle \Theta ({x}_{i})\ln ({p}_{{\xi }_{i}}(\theta ))+(1-\Theta ({x}_{i}))\ln (1-{p}_{{\xi }_{i}}(\theta ))\right\rangle }_{{q}_{\phi }({\boldsymbol{z}}| {\boldsymbol{x}})}.\end{array}$$

In Eq. (7), we use the Gumbel trick for the hits vector. We explain the approach in what follows.

The second term in the r.h.s. of Eq. (3) is known as the VAE regularizer. This term can be parsed as:

8 $${\left\langle \ln \frac{{q}_{\phi }({\boldsymbol{z}}| {\boldsymbol{x}})}{{p}_{\theta }({\boldsymbol{z}})}\right\rangle }_{{q}_{\phi }({\boldsymbol{z}}| {\boldsymbol{x}})}={\langle \ln {q}_{\phi }({\boldsymbol{z}}| {\boldsymbol{x}})\rangle }_{{q}_{\phi }({\boldsymbol{z}}| {\boldsymbol{x}})}-{\langle \ln {p}_{\theta }({\boldsymbol{z}})\rangle }_{{q}_{\phi }({\boldsymbol{z}}| {\boldsymbol{x}})}$$

Let us focus on the first term on the r.h.s. of Eq. (8). When taking the gradient of the entropy with respect to the model parameters one faces two issues: (i) due to the discrete nature of the latent variables *z*, the gradient becomes singular; and (ii) given a function evaluated on a random variable, it is ill-defined taking the gradient of the function with respect to the parameters of the random variable’s probability density function. Moreover, taking the gradient of a discrete estimator, ${\sum }_{{\boldsymbol{z}} \sim {q}_{\phi }({\boldsymbol{z}}| {\boldsymbol{x}})}(...)$, with respect to *ϕ* is not well-defined, since the gradient variables appear in the summation argument. The first issue can be easily addressed by simply considering a continuous step-like function, such as a sigmoid, *σ*(•). Specifically, we replace ***z*** with ***ζ***, where ${\zeta }_{i}=\sigma ({\left[\text{encoded data point}\right]}_{i}\cdot \beta )$, and *β* is an annealing parameter, such that $\mathop{\lim }\limits_{\beta \to \infty }{\boldsymbol{\zeta }}={\boldsymbol{z}}$. The second issue can be addressed by means of the so-called Gumbel trick^47^. The Gumbel trick has become an umbrella term which refers to a set of methods to sample from discrete probabilities or to estimate its partition function. In our case, we simply generate latent variables ***ζ*** via

9 $${\zeta }_{i}=\sigma (({l}_{i}(\phi ,x)+{\sigma }^{-1}({\rho }_{i}))\beta )\quad \,{\rm{for}}\, {\rm{all}}\,i=1,...,m\,,$$

where ${\{{\rho }_{i}\}}_{i = 1}^{m}$ is a set of i.i.d. uniform random numbers, and ${\{{l}_{i}(\phi ,{\boldsymbol{x}})\}}_{i = 1}^{m}$ is a set of logits, i.e., a set of unbounded real numbers generated from the encoder. The connection with Gumbel distributed random numbers is due to the fact that *σ*^−1^(*ρ*) ~ *G*_1_ − *G*_2_, where *G*_1_ and *G*_2_ are two Gumbel distributed random numbers. Moreover, under the recipe given in Eq. (9), one is guaranteed that in the discrete regime of ***ζ*** (i.e., *β* → *∞*), *P*(*ζ*_*i*_ = 1) = *σ*(*l*_*i*_(*ϕ*, *x*))^47–49^. Taking into account the previous, we can then express the entropy as

10 $$\begin{array}{l}{\langle \ln {q}_{\phi }({\boldsymbol{\zeta }}| {\boldsymbol{x}})\rangle }_{{q}_{\phi }({\boldsymbol{z}}| {\boldsymbol{x}})}=\left\langle \mathop{\sum }\limits_{i=1}^{m}\zeta ({l}_{i},\beta ,{\rho }_{i})\ln \sigma ({l}_{i})\right.\\ {\left.+(1-\zeta ({l}_{i},\beta ,{\rho }_{i}))\ln (1-\sigma ({l}_{i}))\right\rangle }_{{q}_{\phi }({\boldsymbol{z}}| {\boldsymbol{x}})}\end{array}$$

The second term in Eq. (8) can be expanded as follows. By design, the functional form of *p*_*θ*_(***z***) is that of a Boltzmann distribution in a heat bath at temperature *T* = 1 (*k*_B_ = 1). Therefore, we have:

11 $${\langle \ln {p}_{\theta }({\boldsymbol{z}})\rangle }_{{q}_{\phi }({\boldsymbol{z}}| {\boldsymbol{x}})}=-{\langle E({\boldsymbol{z}})\rangle }_{{q}_{\phi }({\boldsymbol{z}}| {\boldsymbol{x}})}-\ln Z\,.$$

The first term in the r.h.s. is the energy function averaged over the approximate posterior, whereas the second term is the free energy, which is independent of the encoded samples. The free energy can be replaced by the internal energy, *U*, minus the entropy, *S*, times the temperature, viz. *U*−*TS*. We use the high-temperature gradient approximation to replace the second term in the r.h.s. of Eq. (11) with the internal energy, which is a common practice in approximating the free energy when training RBMs. In the high-temperature gradient approximation, as the energy in the system saturates, the specific heat converges to zero. We demonstrate in Appendix D that when 〈*E*(***z***)〉〈∂*E*(***z***)/∂*ϕ*〉 = 〈*E*(***z***)∂*E*(***z***)/∂*ϕ*〉, the specific heat is zero, which allows us to rewrite the previous equation as

12 $${\langle \ln {p}_{\theta }(z)\rangle }_{{q}_{\phi }({\boldsymbol{z}}| {\boldsymbol{x}})}=-{\langle E({\boldsymbol{z}})\rangle }_{{q}_{\phi }({\boldsymbol{z}}| {\boldsymbol{x}})}+U\,.$$

In this regime, the entropy is solely configurational, scales linearly with the number of units in the RBM, and is independent of the energy parameters.

Computing *U* in Eq. (12) requires calculating the partition function, which becomes intractable beyond a few tens of nodes due to the exponential scaling of the number of states with the number of nodes. Instead, we estimate *U* using uncorrelated samples. To obtain these uncorrelated samples, one performs Markov Chain Monte Carlo simulations, also known as block Gibbs sampling. In the following section, we elaborate on how we sample from the 4-partite RBM.

### Four-partite restricted Boltzmann machine

Let us consider a four-partite restricted Boltzmann machine. For this purpose, we denote each of the four layers as *v*, *h*, *s*, and *t*. The energy function is defined by:

13 $$\begin{array}{rcl}E({\bf{v}},{\bf{h}},{\bf{s}},{\bf{t}})&=&-{a}_{i}{v}_{i}-{b}_{i}{h}_{i}-{c}_{i}{s}_{i}-{d}_{i}{t}_{i}\\ &&-{v}_{i}{W}_{ij}^{(0,1)}{h}_{j}-{v}_{i}{W}_{ij}^{(0,2)}{s}_{j}\\ &&-{v}_{i}{W}_{ij}^{(0,3)}{t}_{j}-{h}_{i}{W}_{ij}^{(1,2)}{s}_{j}\\ &&-{h}_{i}{W}_{ij}^{(1,3)}{t}_{j}-{s}_{i}{W}_{ij}^{(2,3)}{t}_{j}\,,\end{array}$$

where we are using the double indices convention for summation.

The Boltzmann distribution has the following form:

14 $$p({\bf{v}},{\bf{h}},{\bf{s}},{\bf{t}})=\frac{\exp (-E({\bf{v}},{\bf{h}},{\bf{s}},{\bf{t}}))}{Z}\,.$$

where *Z* is the partition function,

15 $$Z=\sum _{\{{\bf{v}},{\bf{h}},{\bf{s}},{\bf{t}}\}}\exp (-E({\bf{v}},{\bf{h}},{\bf{s}},{\bf{t}}))\,.$$

Similar to the two-partite RBM, we can express the distribution over any one of the layers conditioned on the remaining three, in terms of the ratio of the Boltzmann distribution and the marginalized distribution over the layer of interest. Without any loss in generality, let us assume the layer of interest is *h*, then the probability distribution on *p*(**h**∣**v**, **s**, **t**) is given by:

16 $$\begin{array}{rcl}p({\bf{h}}| {\bf{v}},{\bf{s}},{\bf{t}})&=&\frac{p({\bf{v}},{\bf{h}},{\bf{s}},{\bf{t}})}{p({\bf{v}},{\bf{s}},{\bf{t}})}\\ &=&\frac{\exp (-E({\bf{v}},{\bf{h}},{\bf{s}},{\bf{t}}))}{\sum _{{\bf{h}}}\exp (-E({\bf{v}},{\bf{h}},{\bf{s}},{\bf{t}}))}\\ &=&\prod _{i}\frac{\exp ({h}_{i}\cdot ({b}_{i}+{B}_{i}))}{1+\exp ({b}_{i}+{B}_{i})}\end{array}$$

with

17 $${B}_{i}={W}_{ji}^{(01)}{v}_{j}+{W}_{ij}^{(12)}{s}_{j}+{W}_{ij}^{(13)}{t}_{j}$$

We can rewrite Eq. (16) in a rather compact form. Furthermore, we do the same for the case where we condition any of the four partitions on the remaining three. We obtain:

18a $$p({\bf{v}}={\bf{1}}| {\bf{h}},{\bf{s}},{\bf{t}})=\prod _{i}\sigma ({a}_{i}+{A}_{i})\,,$$

18b $$p({\bf{h}}={\bf{1}}| {\bf{v}},{\bf{x}},{\bf{y}})=\prod _{i}\sigma ({b}_{i}+{B}_{i})\,,$$

18c $$p({\bf{s}}={\bf{1}}| {\bf{v}},{\bf{h}},{\bf{t}})=\prod _{i}\sigma ({c}_{i}+{C}_{i})\,,$$

18d $$p({\bf{t}}={\bf{1}}| {\bf{v}},{\bf{h}},{\bf{s}})=\prod _{i}\sigma ({d}_{i}+{D}_{i})\,.$$

where

19a $${A}_{i}={W}_{ij}^{(01)}{h}_{j}+{W}_{ij}^{(02)}{s}_{j}+{W}_{ij}^{(03)}{t}_{j}\,,$$

19b $${B}_{i}={W}_{ji}^{(01)}{v}_{j}+{W}_{ij}^{(12)}{s}_{j}+{W}_{ij}^{(13)}{t}_{j}\,,$$

19c $${C}_{i}={W}_{ji}^{(02)}{v}_{j}+{W}_{ji}^{(12)}{h}_{j}+{W}_{ij}^{(23)}{t}_{j}\,,$$

19d $${D}_{i}={W}_{ji}^{(03)}{v}_{j}+{W}_{ji}^{(13)}{h}_{j}+{W}_{ji}^{(23)}{s}_{j}\,.$$

We then propose a Gibbs sampling process to sample from the joint distribution *p*(**v**, **h**, **s**, **t**) of the four-partite RBM. Notice that the joint distribution can be factorized as *p*(**v**, **h**, **s**, **t**) = *p*(**v**∣**h**, **s**, **t**)*p*(**h**, **s**, **t**). Extending the approach used in two-partite RBM, we perform Markov chain Monte Carlo by sequentially updating each partition conditioned on the remaining, i.e., the prior in step *n* + 1 is deemed the posterior in step *n*. The Gibbs sampling process, at iteration *n*, is done in the following steps:

1. Sample partition ***v***: *p*(***v***∣***h***^(*n*)^, ***s***^(*n*)^, ***t***^(*n*)^)
2. Sample partition ***h***: *p*(***h***∣***v***^(*n*+1)^, ***s***^(*n*)^, ***t***^(*n*)^)
3. Sample partition ***s***: *p*(***s***∣***v***^(*n*+1)^, ***h***^(*n*+1)^, ***t***^(*n*)^)
4. Sample partition ***t***: *p*(***t***∣***v***^(*n*+1)^, ***h***^(*n*+1)^, ***s***^(*n*+1)^)

Notice that one block Gibbs sampling step corresponds to four sampling steps. By repeating this process iteratively, we generate samples that approximate the joint distribution.

We have shown how to generalize an RBM to a four-partite graph. It is important to emphasize that, as with any deep generative model, the features of the dataset are expressed in the latent space. In this context, each state in the latent space encodes specific features of the dataset. Different techniques such as t-SNE^50^ and LSD^51^, can illustrate how dataset features are embedded in latent space after training. These methods allow for the conditioning of the prior on specific features. There is a rather straightforward way to condition the *prior* on given dataset parameters during training, which is widely used across different frameworks. Generally, this involves using specific channels to condition the prior, and specifics depend on the actual framework. An accurate and robust method to sample from the latent space constrained to specific features is crucial for practical applications in generative deep models. In the present context, a calorimeter surrogate requires input related to particle type, incident particle energy, and incidence angle, among other parameters. In the following, we show how to condition the four-partite RBM sampling process.

#### Conditioned four-partite restricted Boltzmann machine

In this section, we outline the approach to condition the four-partite RBM. In this context, the conditioned RBM refers to the scenario where a subset of nodes in the RBM is kept fixed during the block Gibbs sampling process. We adapt the methodology from ref. ^52^ to our Calo4pQVAE. A crucial element of our approach is the use of one complete partition to deterministically encode the data features. Although utilizing the entire partition for feature encoding might appear excessive, it is necessary due to the sparsity of the quantum annealer. Exploring the optimal number of nodes required to efficiently encode the features is beyond the scope of this paper. Moreover, as we move forward towards more complex datasets, we anticipate an increase in the number of features that need to be encoded, underscoring the importance of our chosen approach as a baseline.

Notice that by treating one partition as a conditioning parameter, we effectively modify the self-biases of the remaining partitions. To fix ideas, we consider partition *v* as the conditioning partition, therefore, we may rewrite Eq. (13) as

20 $$\begin{array}{l}E({\bf{h}},{\bf{s}},{\bf{t}}| {\bf{v}})=-({b}_{i}+{v}_{j}{W}_{ji}^{(0,1)}){h}_{i}-({c}_{i}+{v}_{j}{W}_{ji}^{(0,2)}){s}_{i}\\ -({d}_{i}+{v}_{j}{W}_{ji}^{(0,3)}){t}_{i}-{h}_{i}{W}_{ij}^{(1,2)}{s}_{j}\\ -{h}_{i}{W}_{ij}^{(1,3)}{t}_{j}-{s}_{i}{W}_{ij}^{(2,3)}{t}_{j}\,.\end{array}$$

The block Gibbs sampling procedure is the same as before via Eqs. (18) although we skip Eq. (18a). The authors in ref. ^52^ caution on the use of contrastive divergence when training a conditioned RBM due to the possibility of vanishing gradient, even when the conditioned RBM might not be fully trained. This happens when mixing times are larger than the number of Gibbs sampling steps. Despite the previous, we use contrastive divergence in our framework and we show how the estimated log-likelihood saturates after 250 epochs. We further elaborate and discuss this approach in the discussion section.

### Quantum annealers

A quantum annealer (QA) is an array of superconducting flux quantum bits with programmable spin-spin couplings and biases with an annealing parameter^53^. The motivation for QAs comes from the adiabatic approximation^54^, which asserts that if a quantum system is in an eigenstate of its Hamiltonian (which describes the total energy of the system), and the Hamiltonian changes slowly enough, then the system will remain in an eigenstate of the Hamiltonian, although the state itself may change (see Appendix F for a formal derivation). QAs were initially thought of as a faster method to find the ground state of complex problems that could be mapped onto a Hamiltonian *H*^55^. This can be done by initializing the system in the ground state of some Hamiltonian *H*_0_, which is easy to prepare both theoretically and experimentally. In addition, by design, the commutator [*H*, *H*_0_] ≠ 0. The QA interpolates between the two Hamiltonians via

21 $$\begin{array}{r}{H}_{QA}=\frac{{\mathcal{A}}(s)}{2}{H}_{0}+\frac{{\mathcal{B}}(s)}{2}H\end{array}$$

with

22 $$\left\{\begin{array}{l}{H}_{0}=-\sum _{i}{\hat{\sigma }}_{x}^{(i)}\quad \\ H=\sum _{i}{\Delta }_{i}{\hat{\sigma }}_{z}^{(i)}+\sum _{i > j}{J}_{ij}{\hat{\sigma }}_{z}^{(i)}{\hat{\sigma }}_{z}^{(j)}\quad \end{array}\right.$$

such that the annealing parameters, ${\mathcal{A}}(s)$ and ${\mathcal{B}}(s)$, are two slowly-varying controllable parameters constrained to ${\mathcal{A}}(0)\gg {\mathcal{B}}(0)$ and ${\mathcal{A}}(1)\ll {\mathcal{B}}(1)$ and *s* ∈ [0, 1]^56^. In practice, quantum annealers have a strong interaction with the environment which lead to thermalization and decoherence. Evidence suggests that the culprit are the *σ*_*z*_ operator which couple to the environment^57^. There has been efforts towards mitigating decoherence via zero noise extrapolation methods^58^.

Thermalization and decoherence are usually unwanted features in quantum systems as it destroys the quantum state. In our case, these features allow us to replace the RBM with the QA. In other words, RBMs are classical simulations of QAs. A non-desired feature in our framework corresponds to system arrest or freeze-out during annealing^59^, akin to glass melts subject to a rapid quench^60^. Similar to glasses, the annealing time and protocol can have a dramatic impact on the end state^61^. It has been shown that the distribution in this freeze-out state can be approximated by a Boltzmann distribution^30^.

**Fig. 8:**
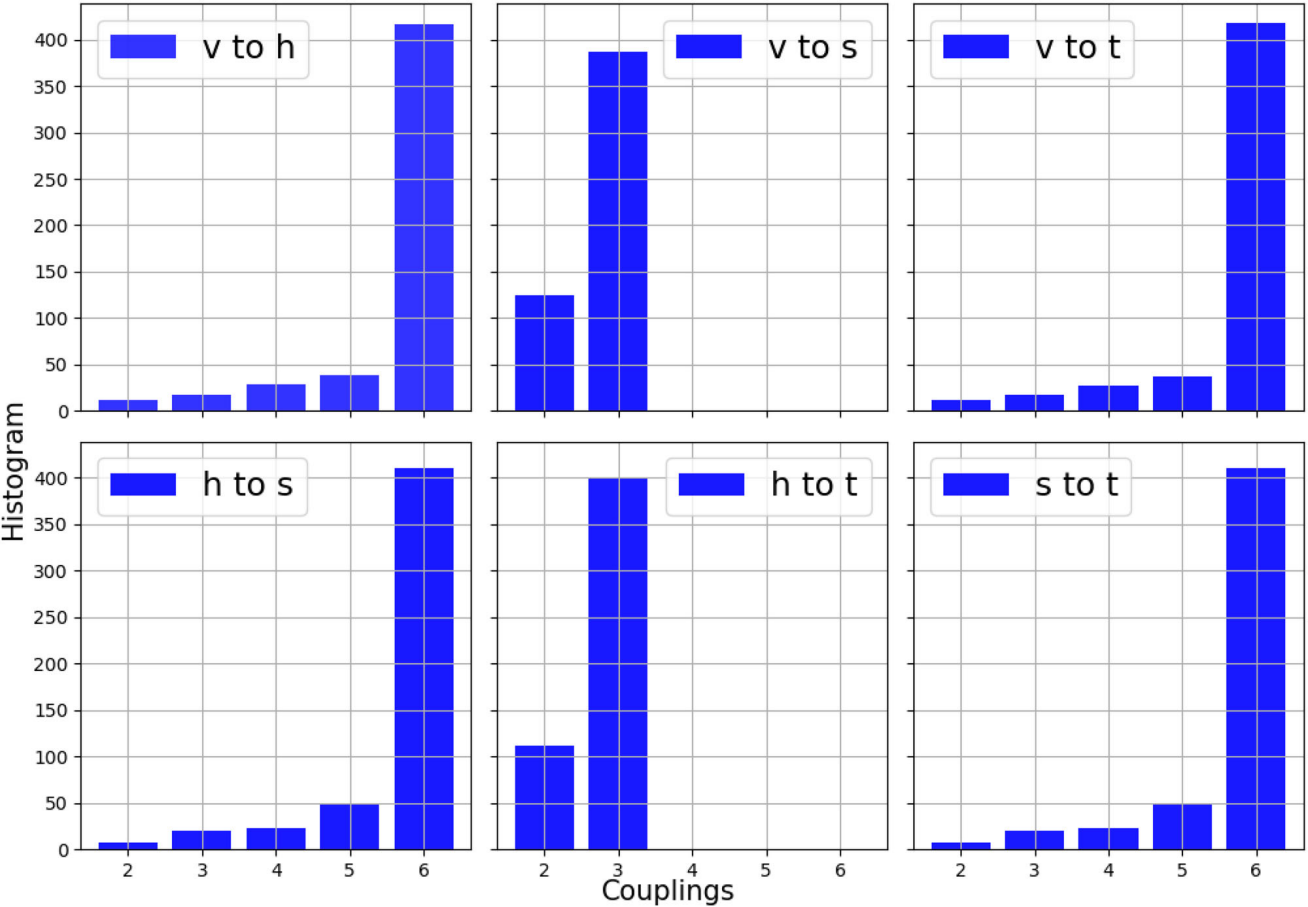
Quadripartite RBM weight matrices. Each panel corresponds to the histogram of connections between partition A and partition B (see legend).

In our pipeline we use Dwave’s *Advantage_system6.4*^62^ which is composed by 5627 qubits and are coupled such that it forms a quadripartite graph. Typically each qubit is coupled with 16 other qubits. In Fig. 8 we show the histogram for number of connections between each of the four partitions.

Notice that the RBM data are binary vectors such that each element can take the value of 0 or 1, whereas the qubits can have values −1 or 1. Hence, to map the RBM onto the QA one redefines the RBM variables ${x}_{i}\to ({\sigma }_{z}^{(i)}+1)/2$ and rearrange the terms in the RBM Hamiltonian to be mapped onto the QA (this is explicitly shown in Appendix F). The previous variable change will lead to an energy offset between the RBM and the QA, which henceforth we neglect. It is important to stress that the QA is in a heat bath with temperature *T*_QA_ ≲ 15 mK^63^, while the annealing parameter has an upper bound ${\mathcal{B}}(1)\approx 5.0\cdot 1{0}^{-24}J$^64^, which implies that the prefactor ${\beta }_{QA}=\frac{{\mathcal{B}}(1)}{2{k}_{B}{T}_{QA}}\approx 12$ while in the RBM by design *β*_RBM_ = 1. To ensure that both, the RBM and the QA describe the same Boltzmann distribution with the same temperature, one can either re-scale the QA Hamiltonian by 1/*β*_*QA*_ or the RBM Hamiltonian by *β*_*QA*_. Currently, there is not a way to measure the QA prefactor, however there are different ways to estimate it^57,65^. In addition, there is no direct way to control this prefactor. The most common way to estimate this prefactor is via the Kullback-Liebler divergence between the QA and the RBM distributions, whereby one introduces a tuning parameter *β* as a prefactor in the RBM distribution which is tuned to minimize the Kullback–Liebler divergence. It is in the process of estimating the *β*_*QA*_ that we also indirectly control the parameter by rescaling the Hamiltonian. The previous can be mathematically expressed as

23 $${\beta }_{t+1}={\beta }_{t}-\eta ({\langle H\rangle }_{QA}-{\langle H\rangle }_{RBM})\,.$$

The previous converges when the average energy from the RBM matches that from the QA, in which case, *β* = *β*_*QA*_. Due to coupling-dependent temperature fluctuations, *β*_*QA*_ is expected to change in the process of training the model. For this reason, the method described by Eq. (23) should be employed, in principle, after each parameter update. Notice that optimizing *β* in Eq. (23) requires samples from the RBM, furthermore, it is the classical RBM temperature which is being tuned to match the distribution of the QA. In our case, we want the opposite, to fit the QA distribution to that of the RBM and not the other way around. To address this point, *H*(*x*) is replaced with an annealed Hamiltonian *H*(*x*, *β*) = *H*(*x*)/*β*, i.e., instead of tuning the temperature in the classical RBM, to match the distribution in the QA, one iteratively rescales the Hamiltonian in the QA to effectively tune the QA’s temperature to match that of the classical RBM. This approach, as already mentioned, is well-known^30,57,65^ and has been proven to be empirically robust yet slow to converge. We therefore propose a different protocol which empirically converges faster than the KL divergence:

24 $${\beta }_{t+1}={\beta }_{t}{\left(\frac{{\langle H\rangle }_{QA}}{{\langle H\rangle }_{RBM}}\right)}^{\delta }\,.$$

Notice that the previous map has a fixed point at *β*_*t*_ = *β*_*QA*_. The condition for a stable fixed point is *λ*(*δ*) < 1, where

25 $$\lambda (\delta )=\left\{\begin{array}{l}| 1+\frac{{\sigma }_{QA}^{2}}{{\langle H\rangle }_{B(1)}}| ,\,\delta =1\quad \\ | 1+\delta \frac{{\sigma }_{QA}^{2}}{{\langle H\rangle }_{QA}}| ,\,\delta \ne 1\,.\quad \end{array}\right.$$

**Fig. 9:**
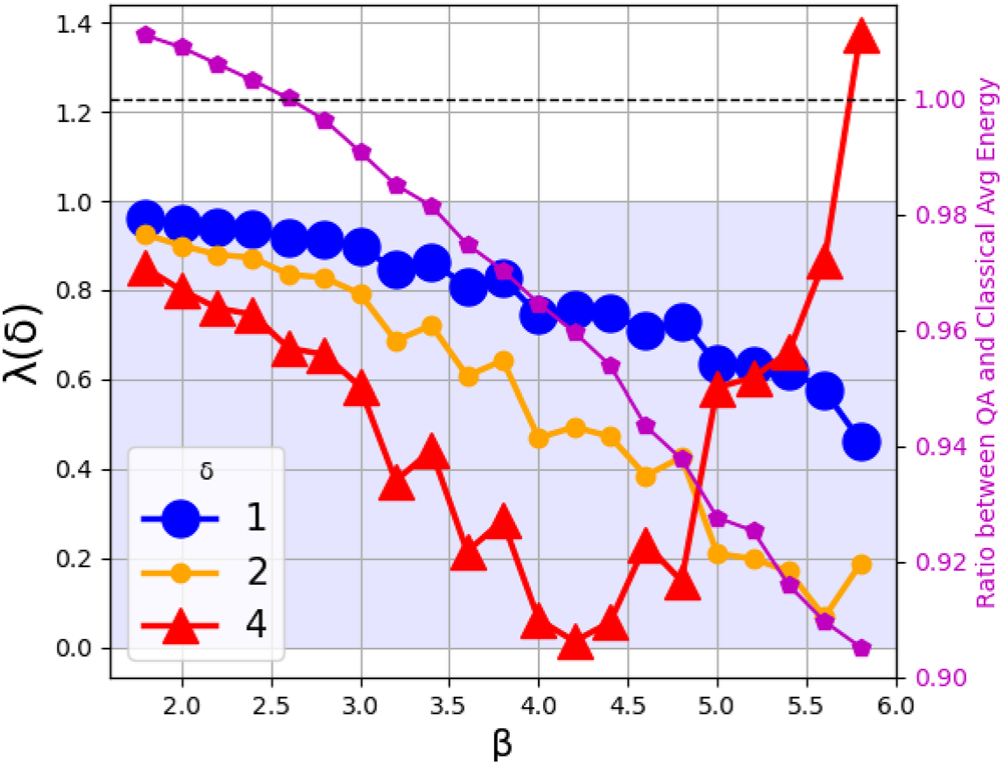
*λ*(*δ*) (see Eq. (25)) vs *β* for different *δ* values (see legend). The stability region is shaded in light blue. Different *δ* values affect the stability depending on the *β* values. For instance, for low *β* values, large *δ* parameters leads to better stability; conversely, large *β* values with large *δ* parameter leads to instabilities. The purple pentagons correspond to the average energy ratio between QPU samples and classical samples. The intersection between the black dashed line and the purple curve defines the fixed point.

In Fig. 9, we show Eq. (25) vs *β* for different values of *δ*. The values of *β* chosen for this plot correspond to where we typically find the fixed point. We call *δ* a stability parameter since we can tune it to stabilize the mapping per iteration and modulate the fixed point attractor feature to ultimately converge in a smaller number of iterations, as we show in the next section. The purple markers in Fig. 9 correspond to the ratio of the average RBM energy obtained from the QA and that obtained from classical sampling. When the ratio equals one, the mapping in Eq. (24) is at the fixed point. In Appendix F we provide a fully detailed derivation and in the Results section we compare both methods.

Up to this point, we have detailed the process of replacing the RBM with the QA in the Calo4pQVAE. As previously mentioned, we utilize a conditioned RBM to enable the sampling of showers with specific characteristics. In the next section, we will explore various approaches for conditioning the QA, aiming to achieve targeted sampling of particle showers with desired features.

#### Conditioned quantum annealer

Quantum annealers were designed to find solutions to optimization problems by identifying a Boolean vector that satisfies a given set of constraints. Typically, QAs are not intended to be conditioned or manipulated in terms of fixing specific qubits during the annealing process. However, there are different approaches that can be employed to fix a set of qubits during the annealing process. In this context, we will present two such approaches. For clarity, let us revisit the concept of conditioning in the realm of QAs. Our goal is to utilize QAs in a manner that allows us to fix a subset of qubits, denoted as ${\sigma }_{z}^{(k)}$ (see Eq. (22)), a priori, such that these qubits remain in their predetermined states throughout and after the annealing process.

##### Reverse annealing with zero transverse field for conditioning qubits

This approach requires control over the biases in *H*_0_ from Eq. (22), where ${H}_{0}={\sum }_{i}{\kappa }_{i}{\hat{\sigma }}_{x}^{(i)}$ and {*κ*_*i*_} are directly specified by the user. Initially, we set the qubits encoding the condition ${\sigma }_{z}^{(k)}$ while the rest of the qubits are randomly initialized. We also set the biases *κ*_*k*_ = 0 to ensure that the transverse field does not alter the state of ${\sigma }_{z}^{(k)}$. Subsequently, we perform reverse annealing, by starting from *s* = 1 and reversing the annealing process towards *s* = 0, before completing the annealing process as usual. The primary drawbacks in this approach are:

- Speedup compromise: The annealing process is effectively doubled in duration due to the reverse annealing step.
- Condition destructed by thermal fluctuations: There is a possibility of the conditioned state being altered due to thermal fluctuations.

Both drawbacks can be mitigated by decreasing the annealing time, as this would not only reduce the overall duration but also minimize the destruction of the conditioned-encoding state by thermal fluctuations. However, as previously noted, reducing the annealing time can lead to the issue of dynamical arrest, where the system becomes trapped in a local minimum^59^. Given that our current framework relies on thermodynamic fluctuations, we leave this approach for future exploration and proceed to discuss an alternative method that does not suffer from these drawbacks and is more practical for immediate implementation.

##### Conditioning qubits through flux biases

This approach relies on using the external flux bias, ${{\rm{\Phi }}}_{k}^{x}$, as effective biases to fix the qubits during annealing. The external flux bias were introduced as a practical remedy to render the biases *h*_*i*_ time-independent during the annealing by having $B(s)h_i=2{{\rm{\Phi }}}_{i}^{x}{I}_{p}(s)$, where *I*_*p*_(*s*) denotes the magnitude of the supercurrent flowing about the rf-SQUID loop. This flux bias is applied to the qubit loop about which the supercurrent flows^66^. Flux biases work as effective biases on qubits. Hence, before the annealing, we specify the encoded incident energy via the flux biases. Throughout our experiments, we always made sure that the partition encoding the incident energy in the QA after annealing matched the encoded incident energy before the annealing.

## Supplementary information

Supplementary information

## Data Availability

The datasets used in this study in publicly available in the repository Fast Calorimeter Simulation Challenge 2022 in https://calochallenge.github.io/homepage/.

## Code availability

The underlying code for this study is publicly available in ref. ^67^.
