# Supplementary material for: Conditioned quantum-assisted deep generative surrogate for particle-calorimeter interactions
Source: npj Quantum Inf. 2025 Jul 7;11(1):114. doi: 10.1038/s41534-025-01040-x (PMC12234362; doi:10.1038/s41534-025-01040-x)
Supplement: Supplementary file 1 — Supplementary information [file 41534_2025_1040_MOESM1_ESM.pdf]

Supplementary Information for CONDITIONED QUANTUM-ASSISTED DEEP GENERATIVE SURROGATE FOR PARTICLE-CALORIMETER INTERACTIONS

J. Quetzalcóatl Toledo-Marín[1, 2,*], Sebastian Gonzalez[1,+], Hao Jia[3,+], Ian Lu[1,+], Deniz Sogutlu[1], Abhishek Abhishek[4], Colin Gay[3], Eric Paquet[4], Roger Melko[2], Geoffrey C. Fox[6], Maximilian Swiatlowski[1], and Wojciech Fedorko[1,*]

[1]TRIUMF, Vancouver, BC V6T 2A3, Canada
[2]Perimeter Institute for Theoretical Physics, Waterloo, Ontario, N2L 2Y5, Canada
[3]Department of Physics and Astronomy, University of British Columbia, Vancouver, BC V6T 1Z1, Canada
[4]Department of Electrical and Computer Engineering, University of British Columbia, Vancouver, BC V6T 1Z4, Canada
[5]Digital Technologies Research Centre, National Research Council, 1200 Montreal Road, Building M-50, Ottawa, Ontario, K1A 0R6, Canada
[6]University of Virginia, Computer Science and Biocomplexity Institute, 994 Research Park Blvd, Charlottesville, Virginia, 22911, USA
[+]These authors contributed equally and are listed in alphabetical order.
[*]Corresponding authors: jtoledo@triumf.ca , wfedorko@triumf.ca.

May 27, 2025

A Variational Autoencoder

In this section we describe the VAE framework first proposed by Kingma and Welling in [1]. Suppose we have a data set $\{\boldsymbol{x}^{(i)}\}_{i=1}^{|\mathscr{D}|}$, where each element in the data set lives in $\mathscr{R}^N$. The goal in training a Variational Autoencoder (VAE) on this data set is to fit a probability distribution, $p(\boldsymbol{x})$, to the data. This is done by maximizing the log-likelihood (LL) of $p(\boldsymbol{x})$ over the data set. A key component in generative models is the introduction of latent variables, $\boldsymbol{z}$, such that the joint distribution can be expressed as $p(\boldsymbol{x},\boldsymbol{z}) = p(\boldsymbol{x}|\boldsymbol{z})p(\boldsymbol{z})$, where $p(\boldsymbol{z})$ is the *prior* distribution of $\boldsymbol{z} \in \mathscr{R}^M$. VAEs are composed by an encoder and a decoder, and are trained using the Evidence Lower Bound (ELBO) as a proxy loss function for the LL. To understand the relationship between the LL and the ELBO, we first write the following identity:

$$\ln p_\theta(\boldsymbol{x}) = \langle \ln p_\theta(\boldsymbol{x}) \rangle_{q_\phi(\boldsymbol{z}|\boldsymbol{x})} \tag{1}$$

where $\langle \bullet \rangle_{q_\phi(\boldsymbol{z}|\boldsymbol{x})}$ denotes expectation value of $\bullet$ over $q_\phi(\boldsymbol{z}|\boldsymbol{x})$. Here, $q_\phi(\boldsymbol{z}|\boldsymbol{x})$ is the encoding function, also known as the approximate posterior. This function encodes the data $\boldsymbol{x}$ into $\boldsymbol{z}$ in the latent space. We can further manipulate the r.h.s. in Eq. (1), *viz.*,

$$\begin{aligned}
\ln p_\theta(\boldsymbol{x}) &= \langle \ln \frac{p_\theta(\boldsymbol{x},\boldsymbol{z})}{p_\theta(\boldsymbol{z}|\boldsymbol{x})} \rangle_{q_\phi(\boldsymbol{z}|\boldsymbol{x})} \\
&= \langle \ln \frac{p_\theta(\boldsymbol{x},\boldsymbol{z}) q_\phi(\boldsymbol{z}|\boldsymbol{x})}{q_\phi(\boldsymbol{z}|\boldsymbol{x}) p_\theta(\boldsymbol{z}|\boldsymbol{x})} \rangle_{q_\phi(\boldsymbol{z}|\boldsymbol{x})} \\
&= \langle \ln \frac{p_\theta(\boldsymbol{x},\boldsymbol{z})}{q_\phi(\boldsymbol{z}|\boldsymbol{x})} \rangle_{q_\phi(\boldsymbol{z}|\boldsymbol{x})} + \langle \ln \frac{q_\phi(\boldsymbol{z}|\boldsymbol{x})}{p_\theta(\boldsymbol{z}|\boldsymbol{x})} \rangle_{q_\phi(\boldsymbol{z}|\boldsymbol{x})} \\
&= \mathscr{L}_{\phi,\theta}(\boldsymbol{x}) + D_{kl}(q_\phi(\boldsymbol{z}|\boldsymbol{x}) || p_\theta(\boldsymbol{z}|\boldsymbol{x})) \,.
\end{aligned} \tag{2}$$

In the last line in the previous Eq., $\mathscr{L}_{\phi,\theta}(\boldsymbol{x})$ is the ELBO and $D_{kl}(q_\phi(\boldsymbol{z}|\boldsymbol{x})||p_\theta(\boldsymbol{z}|\boldsymbol{x}))$ is the Kullback-Liebler (KL) divergence. The KL divergence is a positive functional and equals zero when both distributions are the same.

Therefore:

$$\mathscr{L}_{\phi,\theta}(\boldsymbol{x}) = \ln p_\theta(\boldsymbol{x}) - D_{kl}(q_\phi(\boldsymbol{z}|\boldsymbol{x})||p_\theta(\boldsymbol{z}|\boldsymbol{x})) \le \ln p_\theta(\boldsymbol{x}) \tag{3}$$

The previous Eq. shows that maximizing the ELBO implies maximizing the LL (since the LL is the upper bound), as well as to minimizing the KL divergence between $q_\phi(\boldsymbol{z}|\boldsymbol{x})$ and $p_\theta(\boldsymbol{z}|\boldsymbol{x})$.

We can express the ELBO in a more tractable way:

$$\begin{aligned}\mathscr{L}_{\phi,\theta}(\boldsymbol{x}) &= \langle \ln \frac{p_\theta(\boldsymbol{x},\boldsymbol{z})}{q_\phi(\boldsymbol{z}|\boldsymbol{x})}\rangle_{q_\phi(\boldsymbol{z}|\boldsymbol{x})} \\ &= \langle \ln p_\theta(\boldsymbol{x}|\boldsymbol{z})\rangle_{q_\phi(\boldsymbol{z}|\boldsymbol{x})} - \langle \ln \frac{q_\phi(\boldsymbol{z}|\boldsymbol{x})}{p_\theta(\boldsymbol{z})}\rangle_{q_\phi(\boldsymbol{z}|\boldsymbol{x})} . \end{aligned} \tag{4}$$

The first term in the last equality is called the reconstruction term since it is a measure of how well the model is able to reconstruct the input $\boldsymbol{x}$ from a latent vector $\boldsymbol{z}$. The second term in the last equality is called a regularizer and measures the divergence between the prior and the approximate posterior.

The legacy VAE [1] assumes the functional forms:

$$p_\theta(\boldsymbol{x}|\boldsymbol{z}) = \prod_{i=1}^{N} \frac{1}{\sqrt{2\pi\sigma^2_{\hat{x}_i}}} \exp\left(-\frac{(x_i - \hat{x}_i)^2}{2\sigma^2_{\hat{x}_i}}\right) \tag{5a}$$

$$p_\theta(\boldsymbol{z}) = \prod_{i=1}^{M} \frac{1}{\sqrt{2\pi}} \exp(z_i^2/2) \tag{5b}$$

$$q_\phi(\boldsymbol{z}|\boldsymbol{x}) = \prod_{i=1}^{M} \frac{1}{\sqrt{2\pi\sigma_i^2}} \exp\left(-\frac{(z_i - \mu_i)^2}{2\sigma_i^2}\right) . \tag{5c}$$

This leads to the following expression for the ELBO:

$$\mathscr{L}_{\phi,\theta}(\boldsymbol{x}) = -\sum_{i=1}^{N} \langle (x_i - \hat{x}_i)^2 \rangle_{q_\phi(z|x)} - \sum_{i=1}^{M} \frac{1}{2}\left(\mu_i^2 + \sigma_i^2 - 1 - \ln \sigma_i^2\right) + \text{const} . \tag{6}$$

To obtain the previous equality we assumed $\boldsymbol{\sigma}_{\hat{x}} = \mathbf{1}$. We also used the fact that $\langle z_i^2 \rangle_{q_\phi} = \mu_i^2 + \sigma_i^2$. While the goal is to maximize the ELBO, it is common practice to minimize the negative ELBO during training. An important step in the context of VAEs is the so-called *reparameterization trick*. When dealing with a finite data set and optimizing the loss function we need to compute gradients of expectations with respect to distributions that may not be explicitly expressible. For instance, consider computing the gradient of an estimator:

$$\nabla_\phi \langle f_\phi(z)\rangle_{q_\phi(z)} = \nabla_\phi \int dz q_\phi(z) f_\phi(z) \tag{7}$$

$$\sim \nabla_\phi \sum_{z\sim q_\phi(z)} f_\phi(z) \tag{8}$$

The issue here is that taking the gradient over $\sum_{z\sim q_\phi(z)}$ is rather ill-defined because the samples are drawn from a distribution parameterized by ϕ. To circumvent this, the reparameterization trick consists in a change in variable in a way that makes the sampling process differentiable with respect to ϕ. This variable change needs to preserve the metric, *i.e.*, the distribution in the old variable and that in the new variable need to both be normalized:

$$\int dz q_\phi(z) = \int d\epsilon \rho(\epsilon) \implies \rho(\epsilon) = |\frac{dz}{d\epsilon}| q_\phi(z) \tag{9}$$

The simplest change in variable is $z = \mu + \sigma\epsilon$ with $\epsilon \sim \mathscr{N}(1,0)$, which leads to

$$\nabla_\phi \sum_{z\sim q_\phi(z)} f_\phi(z) \to \sum_{\epsilon\sim\mathscr{N}(0,1)} \nabla_\phi f_\phi(\mu_\phi + \sigma_\phi \epsilon) \tag{10}$$

The *reparameterization trick* is merely a change in variable akin to that used in the Box-Muller method to generate Gaussian distributed random numbers from Uniform distributed random numbers [2]. This is quite often used in the context of deep generative models in order to be able to take the gradient over an estimator.

B Discrete Variational Autoencoder

Discrete Variational Autoencoders (DVAEs) are a type of VAE where the latent space is discrete. The main two challenges with DVAEs are *i*) how does one backpropagate the gradient since the latent space is discrete? *ii*) what reparameterization can be employed to enable gradient-based optimization? To address the former, one can simply relax the discrete condition by introducing annealed sigmoids. Specifically, we replace the Heaviside function $\Theta(x)$ with the sigmoid function $\sigma(x\beta)$, where β is the annealing parameter. Notice that $\lim_{\beta\to\infty}\sigma(x\beta) = \Theta(x)$. To address the latter issue one can employ the *Gumbel trick*. The *Gumbel trick* has become an umbrella term which refers to a set of methods to sample from discrete probabilities or to estimate its partition function. In our case, we simply generate latent variables ζ via

$$\zeta = \sigma((l(\phi, x) + \sigma^{-1}(\rho))\beta)\,, \tag{11}$$

where ρ is a uniform random number, and $l(\phi, x)$ is a logit, *i.e.*, the inverse of a sigmoid function, such that in the discrete regime of ζ (*i.e.*, $\beta \to \infty$) $P(\zeta = 1) = \sigma(l(\phi, x))$. Notice that in this approach, we generate the random variable ζ using a deterministic equation, σ; a logit, $l(\phi, x)$; and a uniformly-distributed random number, ρ. The connection with Gumbel distributed random numbers is due to the fact that $\sigma^{-1}(\rho) \sim G_1 - G_2$, where G_1 and G_2 are two Gumbel distributed random numbers [3, 4, 5].

C Bipartite Restricted Boltzmann Machines

Suppose a data set $\{\boldsymbol{v}^{(i)}\}_{i=1}^{|\mathscr{D}|}$, and each element in the data set lives in $\{0,1\}^N$. The goal behind training a Restricted Boltzmann Machine (RBM) over this data set consists on fitting a probability mass function, $p(\boldsymbol{v})$, that models the distribution of the data. This is achieved by maximizing the log-likelihood (LL) of $p(\boldsymbol{v})$ over the data set. We denote the joint probability of the dataset as $P_{\mathscr{D}} = \left(\prod_{\boldsymbol{v}\in\mathscr{D}} p(\boldsymbol{v})\right)^{1/|\mathscr{D}|}$. Maximizing the LL corresponds to:

$$\boldsymbol{\Omega}\ln P_{\mathscr{D}} \tag{12}$$

By design, $p(\boldsymbol{v})$ follows a Boltzmann distribution *viz.*

$$p(\boldsymbol{v}) = \frac{\sum_{\boldsymbol{h}} e^{-E(\boldsymbol{v},\boldsymbol{h};\boldsymbol{a},\boldsymbol{b},\boldsymbol{W})}}{Z(\boldsymbol{a},\boldsymbol{b},\boldsymbol{W})}\,, \tag{13}$$

where $Z(\boldsymbol{a},\boldsymbol{b},\boldsymbol{W})$ is the partition function and $E(\boldsymbol{v},\boldsymbol{h};\boldsymbol{a},\boldsymbol{b},\boldsymbol{W})$ is the energy function defined as

$$E(\boldsymbol{v},\boldsymbol{h};\boldsymbol{a},\boldsymbol{b},\boldsymbol{W}) = -\sum_i a_i v_i - \sum_j b_j h_j - \sum_{i,j} v_i W_{ij} h_j\,. \tag{14}$$

The parameters $\boldsymbol{a}$, $\boldsymbol{b}$ and $\boldsymbol{W}$ are fitting parameters and $\boldsymbol{h}$ is called the hidden vector such that $\boldsymbol{h} \in \{0,1\}^M$. Notice that the matrix $\boldsymbol{W}$ couples the nodes in $\boldsymbol{v}$ with the nodes in $\boldsymbol{h}$, while there are no explicit couplings between nodes in $\boldsymbol{v}$ nor between nodes in $\boldsymbol{h}$, which is the same to say that the RBM is a bipartite graph.

Notice that

$$\frac{\partial E}{\partial w} = \begin{cases} -v_k & w = a_k, \\ -h_k & w = b_k, \\ -v_k h_l & w = W_{kl}\,. \end{cases} \tag{15}$$

Taking the derivative of the LL with respect to some generic parameter w yields:

$$\frac{\partial \ln P_{\mathscr{D}}}{\partial w} = \frac{1}{|\mathscr{D}|}\sum_{i=1}^{|\mathscr{D}|}\langle -\frac{\partial E}{\partial w}\rangle_{p(\boldsymbol{h}|\boldsymbol{v}^{(i)})} - \langle -\frac{\partial E}{\partial w}\rangle_{p(\boldsymbol{v},\boldsymbol{h})} \tag{16}$$

The previous simplifies to

$$\frac{\partial \ln P_{\mathscr{D}}}{\partial a_k} = \frac{1}{|\mathscr{D}|}\sum_{i=1}^{|\mathscr{D}|}\langle v_k\rangle_{p(\boldsymbol{h}|\boldsymbol{v}^{(i)})} - \langle v_k\rangle_{p(\boldsymbol{v},\boldsymbol{h})} \tag{17a}$$

$$\frac{\partial \ln P_{\mathscr{D}}}{\partial b_k} = \frac{1}{|\mathscr{D}|}\sum_{i=1}^{|\mathscr{D}|}\langle h_k\rangle_{p(\boldsymbol{h}|\boldsymbol{v}^{(i)})} - \langle h_k\rangle_{p(\boldsymbol{v},\boldsymbol{h})} \tag{17b}$$

$$\frac{\partial \ln P_{\mathscr{D}}}{\partial w_{kl}} = \frac{1}{|\mathscr{D}|}\sum_{i=1}^{|\mathscr{D}|}\langle v_k h_l\rangle_{p(\boldsymbol{h}|\boldsymbol{v}^{(i)})} - \langle v_k h_l\rangle_{p(\boldsymbol{v},\boldsymbol{h})} \tag{17c}$$

where

$$\langle \bullet \rangle_{p(\boldsymbol{h}|\boldsymbol{v}^{(i)})} = \frac{\sum_{\boldsymbol{h}} \bullet e^{-E(\boldsymbol{v}^{(i)},\boldsymbol{h})}}{\sum_{\boldsymbol{h}} e^{-E(\boldsymbol{v}^{(i)},\boldsymbol{h})}} \tag{18}$$

and

$$\langle \bullet \rangle_{p(\boldsymbol{v},\boldsymbol{h})} = \frac{\sum_{\boldsymbol{v},\boldsymbol{h}} \bullet e^{-E(\boldsymbol{v},\boldsymbol{h})}}{\sum_{\boldsymbol{v},\boldsymbol{h}} e^{-E(\boldsymbol{v},\boldsymbol{h})}} \,. \tag{19}$$

Since the number of $\boldsymbol{v}$ and $\boldsymbol{h}$ states are 2^N and 2^M, respectively, the number of terms in the summations in Eqs. (18) and (19) are $|\mathscr{D}| \times 2^M$ and 2^{N+M}, respectively. This exponential dependence on the dimensionality makes computing these averages intractable. To overcome this challenge, importance sampling is employed.

Notice that $q(\boldsymbol{h}|\boldsymbol{v}) = p(\boldsymbol{v},\boldsymbol{h})/p(\boldsymbol{v})$, from which it is straightforward showing

$$q(\boldsymbol{h}|\boldsymbol{v}) = \prod_{j=1}^{M} q(h_j|\boldsymbol{v})\,, \tag{20}$$

where

$$q(h_j|\boldsymbol{v}) = \frac{e^{h_j C_j(\boldsymbol{v})}}{1+e^{C_j(\boldsymbol{v})}} \tag{21}$$

and $C_j(\boldsymbol{v}) = \sum_i v_i W_{ij} + b_j$. Therefore, $q(h_j = 1|\boldsymbol{v}) = \sigma(C_j(\boldsymbol{v}))$. Conversely, $p(v_i = 1|\boldsymbol{h}) = \sigma(D_i(\boldsymbol{h}))$, with $D_i(\boldsymbol{h}) = \sum_j W_{ij} h_j + a_i$. Hence, we can employ the expressions $\sigma(D_i(\boldsymbol{h}))$ and $\sigma(C_j(\boldsymbol{v}))$ to perform Gibbs sampling.

We can further simplify the expectation values from Eq. (18):

$$\langle v_k \rangle_{p(\boldsymbol{h}|\boldsymbol{v}^{(i)})} = v_k \tag{22a}$$

$$\langle h_j \rangle_{p(\boldsymbol{h}|\boldsymbol{v}^{(i)})} = \frac{e^{C_j(\boldsymbol{v})}}{1+e^{C_j(\boldsymbol{v})}} \tag{22b}$$

$$\langle v_k h_j \rangle_{p(\boldsymbol{h}|\boldsymbol{v}^{(i)})} = v_k \frac{e^{C_j(\boldsymbol{v})}}{1+e^{C_j(\boldsymbol{v})}} \tag{22c}$$

$$\tag{22d}$$

Note that $p(\boldsymbol{v},\boldsymbol{h}) = p(\boldsymbol{v}|\boldsymbol{h})p(\boldsymbol{h})$ and $p(\boldsymbol{v},\boldsymbol{h}) = q(\boldsymbol{h}|\boldsymbol{v})p(\boldsymbol{v})$. Therefore, starting from an observed data point, $\boldsymbol{v}$, we can generate samples of the hidden units, $\boldsymbol{v}$, via $p(\boldsymbol{h}|\boldsymbol{v})$. These samples can then be used as prior samples to generate new samples of $\boldsymbol{v}$ vectors. We can repeat the process a number of times K, called the number of Gibbs sampling steps. We denote this process as $(\boldsymbol{v},\boldsymbol{h}) \sim \left[q(\boldsymbol{h}|\boldsymbol{v})p(\boldsymbol{v}|\boldsymbol{h})\right]^K$

Therefore, for very large K, we can estimate Eq. (19) as:

$$\langle \bullet \rangle_{p(\boldsymbol{v},\boldsymbol{h})} \simeq \frac{1}{N} \sum_{(v,h)\sim\left[q(h|v)p(v|h)\right]^K} \bullet \,. \tag{23}$$

The Gibbs sampling number of steps ultimately should be larger than the mixing time. The mixing time will depend on the size of the RBM, the data set being used and, interestingly, it has been shown that as the number of updates during training increases, the Gibbs sampling number of steps must increase for the RBM to reach equilibrium and avoid getting stuck in an non-equilibrium state [6].

The standard procedure to train an RBM involves partitioning the data set $\mathscr{D}$ into mini-batches $\mathscr{D}_\alpha$, such that $\mathscr{D} = \cup_\alpha \mathscr{D}_\alpha$. Then the RBM parameters are updated by:

$$a_k^{(t)} = a_k^{(t-1)} + \eta \frac{\partial \ln P_{\mathscr{D}_\alpha}}{\partial a_k}\,, \tag{24a}$$

$$b_k^{(t)} = b_k^{(t-1)} + \eta \frac{\partial \ln P_{\mathscr{D}_\alpha}}{\partial b_k}\,, \tag{24b}$$

$$W_{kl}^{(t)} = W_{kl}^{(t-1)} + \eta \frac{\partial \ln P_{\mathscr{D}_\alpha}}{\partial W_{kl}}\,, \tag{24c}$$

where η is the learning rate. When performing importance sampling, it is common to generate Markov chains of the size of the mini-batch, $|\mathscr{D}_\alpha|$.

There are three primary methods for training RBMs in the literature. Each one mainly differs from the others in the manner in which the Markov chains are initialized. The simplest one correspond to the case where for each parameter update, the initial state is randomly sampled from a 1/2-Bernoulli distribution and is called *Rdm-K*, where K is the number for Gibbs sampling steps. Another way shown to yield more robust RBMs is called *Contrastive Divergence* (CD), whereby the Markov chain is initialized from a point in the dataset. Lastly, persistent contrastive divergence (PCD) is very similar to CD in the sense that the Markov chain is started using a data point in the data set for the first parameter update, while for the remaining parameter updates, the Markov chains are initialized using the last state in the previous parameter update. This is similar to the traditional way to sample from an Ising model when decreasing the temperature. Instead of restarting the Markov chain from a random state after each temperature update, the chain is restarted from the previous state before the temperature update.

D High temperature gradient approximation

The previous section shows the derivation of the block Gibbs sampling Eqs. used to trained RBM. A quite common approach to training RBMs consists in replacing $\ln Z$ with the average energy before computing the gradient. The basis comes from noticing that the gradient of the logarithm of the partition function w.r.t. the RBM parameters is equal to the average value of the gradient of the energy w.r.t. the RBM parameters, *viz.*,

$$-\frac{\partial \ln Z}{\partial \phi} = \langle \frac{\partial E}{\partial \phi} \rangle \tag{25}$$

This expectation value is over the ensemble and it is approximated by an arithmetic average over samples obtained via block Gibbs sampling, *i.e.*,

$$\langle \frac{\partial E}{\partial \phi} \rangle \simeq \frac{1}{N} \sum_{z \sim BGS} \frac{\partial E}{\partial \phi} = \frac{\partial}{\partial \phi} \frac{1}{N} \sum_{z \sim BGS} E(z) \,. \tag{26}$$

The last equality is not general and only holds in certain scenarios, as we show here. In the following we show that this approximation corresponds to the high temperature gradient approximation where thermal energy is larger than typical spin interactions, such that the specific heat is zero and the only contribution to the entropy is configurational.

By definition, the average energy is given by $\langle E(z) \rangle = \sum_z E(z) e^{-\beta E(z)} / Z(\beta)$. Deriving the energy w.r.t. some parameter ϕ the energy depends on leads to:

$$\frac{\partial}{\partial \phi} \langle E(z) \rangle = \langle \frac{\partial E(z)}{\partial \phi} \rangle + \beta \left(\langle E(z) \rangle \langle \frac{\partial E(z)}{\partial \phi} \rangle - \langle E(z) \frac{\partial E(z)}{\partial \phi} \rangle \right) \tag{27}$$

Hence $\frac{\partial}{\partial \phi} \langle E(z) \rangle = \langle \frac{\partial E(z)}{\partial \phi} \rangle$ implies the equality:

$$\langle E(z) \rangle \langle \frac{\partial E(z)}{\partial \phi} \rangle - \langle E(z) \frac{\partial E(z)}{\partial \phi} \rangle = 0 \,. \tag{28}$$

Recall the specific heat relates to the second cumulant via $C_T = \frac{1}{kT^2} \sigma_E^2$. Notice that C_T depends on ϕ. The derivative of the second cumulant w.r.t. ϕ leads to

$$\frac{\partial \sigma_E^2}{\partial \phi} = \beta \langle \frac{\partial E(z)}{\partial \phi} \rangle \left(\langle E(z)^2 \rangle - 2 \langle E(z) \rangle^2 \right) + 2 \left(\langle E(z) \frac{\partial E(z)}{\partial \phi} \rangle - \langle E(z) \rangle \langle \frac{\partial E(z)}{\partial \phi} \rangle \right) \tag{29}$$

$$-\beta \left(\langle E(z)^2 \frac{\partial E(z)}{\partial \phi} \rangle - 2 \langle E(z) \rangle \langle E(z) \frac{\partial E(z)}{\partial \phi} \rangle \right) \tag{30}$$

From the previous equation it is easy to show that when Eq. (28) is satisfied, $\frac{\partial C_T}{\partial \phi} = 0$. In general, the previous occurs at very large temperatures where $C_T = 0$, *i.e.*, the energy of the system saturates such that increasing the temperature does not increase the energy. In such regime, the spins are uncorrelated and the entropy is solely defined by the logarithm of possible configurations.

E Quadripartite RBM numerical verification

We considered 4-partite RBM with six nodes per partition. This setting allows for the explicit enumeration of all feasible states, facilitating the precise computation of the partition function. To rigorously assess the accuracy of our approach, we conducted a comparative analysis of the density of energy states. This entailed a direct comparison

between the utilization of all feasible states and the implementation of the 4-partite Gibbs sampling method, as elaborated upon in Methods section. In Figure E1, we present a detailed visual comparison of the density of states obtained through both methodologies, across various iterations of the Gibbs sampling process. This comparison shows the convergence and consistency of these two approaches.

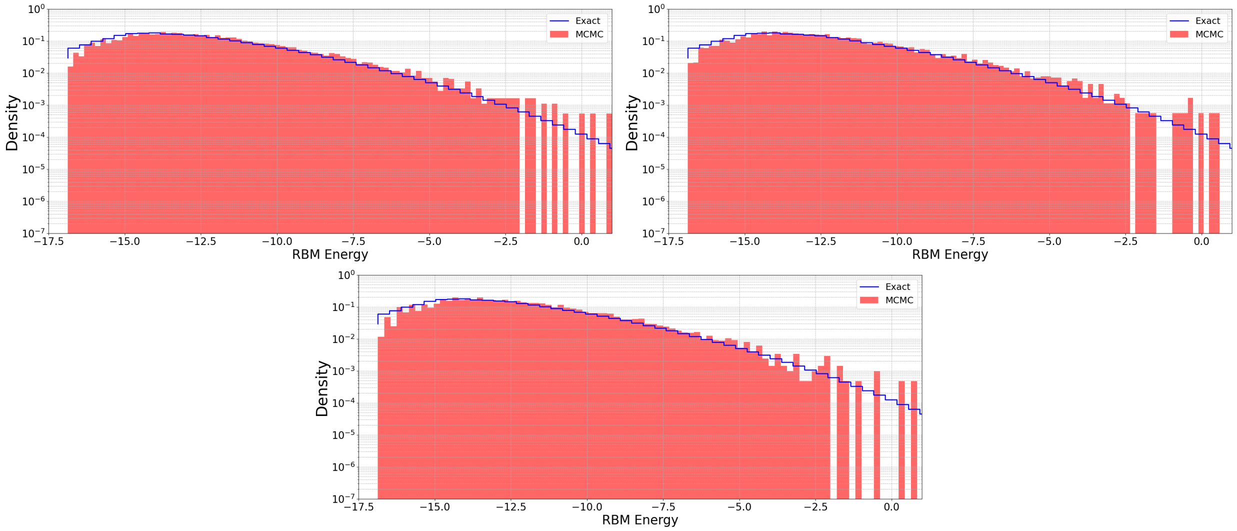

Figure E1: Density of states for 4-partite RBMs with 6×4 nodes and $N = 10240$ samples, with Gibbs sampling steps set to **(upper left panel)** 200, **(upper right panel)** 500, and **(lower panel)** 3000 units.

F Quantum Annealers

F.1 Adiabatic Approximation

Here we derive the adiabatic approximation following [7], which is the theoretical foundation of quantum annealers. Let us suppose a time-dependent Hamiltonian $H(t)$, we denote the time-dependent eigenstates as $|n;t\rangle$ and the eigenvalues as $E_n(t)$, such that,

$$H(t)|n;t\rangle = E_n(t)|n,t\rangle\ , \tag{31}$$

which simply states that at any particular time t, the eigenstate and eigenvalue may change. Notice that one can write the general solution to Schrödinger's Eq., *viz.*,

$$i\hbar\frac{\partial}{\partial t}|\alpha;t\rangle = H(t)|\alpha;t\rangle \tag{32}$$

as

$$|\alpha;t\rangle = \sum_n c_n(t)e^{i\theta(t)}|n;t\rangle \tag{33}$$

with

$$\theta_n(t) = -\frac{1}{\hbar}\int_0^t E_n(t')dt'\ . \tag{34}$$

By substituting Eqs. (33) and (34) in Eq. (32) we reach the following equality:

$$\sum_n e^{i\theta_n(t)}\left[\dot{c}_n(t)|n;t\rangle + c_n(t)\frac{\partial}{\partial t}|n;t\rangle\right] = 0 \tag{35}$$

By taking the inner product w.r.t. $\langle m;t|$ and invoking orthonormality, yields the following differential equation for the time-dependent coefficients:

$$\dot{c}_m(t) = -\sum_n c_n(t)e^{i(\theta_n(t)-\theta_m(t))}\langle m;t|\frac{\partial}{\partial t}|n;t\rangle\ . \tag{36}$$

To get a better sense of the $\langle m;t|\frac{\partial}{\partial t}|n;t\rangle$ term, let us take the time derivative of the characteristic Eq. (31):

$$\begin{aligned}
\langle m;t|\frac{\partial}{\partial t}\left[H(t)|n;t\rangle = E_n(t)|n,t\rangle\right] \implies \\
\langle m;t|\left[\dot{H}(t)|n;t\rangle + H(t)\frac{\partial}{\partial t}|n;t\rangle = \dot{E}_n(t)|n;t\rangle\right. \\
\left.+E_n(t)\frac{\partial}{\partial t}|n;t\rangle\right] \implies \\
\langle m;t|\dot{H}(t)|n;t\rangle = \left(E_n(t) - E_m(t)\right)\langle m;t|\frac{\partial}{\partial t}|n;t\rangle
\end{aligned} \tag{37}$$

(38)

For $m \neq n$, we can write

$$\langle m;t|\frac{\partial}{\partial t}|n;t\rangle = \frac{\langle m;t|\dot{H}(t)|n;t\rangle}{E_n(t)-E_m(t)}\,. \tag{39}$$

Substituting the previous equation in Eq. (36) leads to:

$$\dot{c}_m(t) = -c_m(t)\langle m;t|\frac{\partial}{\partial t}|m;t\rangle \tag{40}$$

$$-\sum_{n\neq m} c_n(t)e^{i(\theta_n(t)-\theta_m(t))}\frac{\langle m;t|\dot{H}(t)|n;t\rangle}{E_n(t)-E_m(t)}\,. \tag{41}$$

The previous Eq. shows that states with $n \neq m$ will mix with $|m;t\rangle$ due to the time dependence of the Hamiltonian.

The adiabatic approximation consists in neglecting the mixing terms which correspond to the regime whereby

$$\frac{|\langle m;t|\dot{H}(t)|n;t\rangle|}{E_n(t)-E_m(t)} \equiv \frac{1}{\tau} \ll \langle m;t|\frac{\partial}{\partial t}|m;t\rangle \sim \frac{E_m}{\hbar}\,. \tag{42}$$

The previous gives us the condition where the adiabatic approximation holds, *i.e.*, that in which the timescale τ for changes in the Hamiltonian is much larger than the inverse of the characteristic frequency of the state phase factor. In such regime,

$$c_n(t) = e^{i\gamma(t)}c_n(0) \tag{43}$$

with

$$\gamma_n(t) \equiv i\int_0^t dt'\langle n;t'|\frac{\partial}{\partial t'}|n;t'\rangle \tag{44}$$

Notice that

$$0 = \frac{\partial}{\partial t}\langle n;t'|n;t'\rangle = \left[\frac{\partial}{\partial t}\langle n;t'|\right]|n;t'\rangle + \langle n;t|\frac{\partial}{\partial t}|n;t\rangle \tag{45}$$

which implies that

$$\left(\langle n;t|\frac{\partial}{\partial t}|n;t\rangle\right)^* = -\langle n;t|\frac{\partial}{\partial t}|n;t\rangle \tag{46}$$

Therefore, the integral argument is imaginary in which case $\gamma(t)$ is real. Hence, in the adiabatic approximation ($\tau \gg 1/\omega_n$), if the system starts out in eigenstate $|n;0\rangle$, it will remain there since $c_n(t) = e^{i\gamma(t)}c_n(0)$ and $c_l(t) = 0$ for all $l \neq n$. Finally, it is important to stress that the adiabatic approximation does not correspond to short time regimes, *i.e.*, the time t is not relevant here but only the Hamiltonian change rate and the characteristic time of the state phase factor.

F.2 Dwave parameter mapping

This subsection shows the explicit parameter mapping between an RBM and a QA. Recall the quadripartite RBM energy function is:

$$\begin{aligned}
E(\mathbf{v},\mathbf{h},\mathbf{s},\mathbf{t}) = -a_i v_i - b_i h_i - c_i s_i - d_i t_i \\
-v_i W^{(0,1)}_{ij} h_j - v_i W^{(0,2)}_{ij} s_j \\
-v_i W^{(0,3)}_{ij} t_j - h_i W^{(1,2)}_{ij} s_j \\
-h_i W^{(1,3)}_{ij} t_j - s_i W^{(2,3)}_{ij} t_j\,,
\end{aligned} \tag{47}$$

where we are using the double indices convention for summation. Since RBM data values are 0s and 1s, while qubits can take the values $\{-1, 1\}$, we map the quantum states to RBM states as:

$$\begin{pmatrix} \mathbf{v} \\ \mathbf{h} \\ \mathbf{s} \\ \mathbf{t} \end{pmatrix} = \frac{1}{2} \begin{pmatrix} \mathbf{z_v} + \mathbf{1} \\ \mathbf{z_h} + \mathbf{1} \\ \mathbf{z_s} + \mathbf{1} \\ \mathbf{z_t} + \mathbf{1} \end{pmatrix} \tag{48}$$

By substituting Eq. (48) in Eq. (47) and reading out the new couplings and biases, we obtain:

$$\begin{aligned}
\Delta_i &= -\frac{a_i}{2} \\
&- \frac{1}{4}\left(\sum_j W^{(01)}_{ij} + W^{(02)}_{ij} + W^{(03)}_{ij}\right), i \in \Phi_0 \\
\Delta_i &= -\frac{b_i}{2} \\
&- \frac{1}{4}\left(\sum_j (W^{(01)})^t_{ij} + W^{(12)}_{ij} + W^{(13)}_{ij}\right), i \in \Phi_1 \\
\Delta_i &= -\frac{c_i}{2} \\
&- \frac{1}{4}\left(\sum_j (W^{(02)})^t_{ij} + (W^{(12)})^t_{ij} + W^{(23)}_{ij}\right), i \in \Phi_2 \\
\Delta_i &= -\frac{d_i}{2} \\
&- \frac{1}{4}\left(\sum_j (W^{(03)})^t_{ij} + (W^{(13)})^t_{ij} + (W^{(23)})^t_{ij}\right), i \in \Phi_3 \\
J_{ij} &= -\frac{W^{(\gamma,\delta)}_{ij}}{4}, i \in \Phi_\gamma \text{ and } j \in \Phi_\delta
\end{aligned} \tag{49}$$

where Φ_i denotes the partition i and $(\bullet)^t$ denotes transpose. In addition, this mapping introduces an energy offset, H_o, in the Hamiltonian

$$H_o = -\left(\frac{1}{2}\sum_i a_i + b_i + c_i + d_i + \frac{1}{4}\sum_j \sum_{\gamma<\delta} W^{(\gamma,\delta)}_{ij}\right), \tag{50}$$

which we ignore since it does not contribute to the state probability distribution. After applying this transformation, we obtain the new RBM Hamiltonian:

$$H_{RBM} = \sum_i \Delta^{RBM}_i z_i + \sum_{ij} J^{RBM}_{ij} z_i z_j \,. \tag{51}$$

F.3 DWave β_{QA} parameter estimation: Method 1

Let us assume two RBMs which we denote as QA and B, both, described by the same Hamiltonian at different temperatures, *viz.*:

$$P_{QA}(x) = \frac{e^{-\beta_{QA} H(x)}}{Z(\beta_{QA})}, \tag{52}$$

$$P_B(x) = \frac{e^{-\beta H(x)}}{Z(\beta)}. \tag{53}$$

We denote as β_{QA} and β the inverse temperatures of system QA and B, respectively. The Kullback-Liebler divergence associated to these two system yields:

$$D_{KL}(P_{QA}||P_B) = (\beta - \beta_{QA})\langle H\rangle_{QA} + \ln\frac{Z(\beta)}{Z(\beta_{QA})}, \tag{54}$$

from which it is trivial to show that $\beta = \beta_{QA}$ yields zero in the KL divergence. The KL divergence derivative w.r.t. β yields

$$\frac{\partial D_{KL}}{\partial \beta} = \langle H \rangle_{QA} - \langle H \rangle_{B(\beta)} \,, \tag{55}$$

where we have made explicit the β dependence of system B. We can fit β through gradient descent using the KL divergence, which leads to:

$$\beta_{t+1} = \beta_t - \eta \left(\langle H(x) \rangle_{QA} - \langle H(x) \rangle_{B(\beta)} \right) \tag{56}$$

The pseudo-algorithm to fit β using Eq. (56) is:

1. Fix learning rate η and initialize β_0. Parse the RBM parameters onto the Quantum Annealer via Eqs. (49).
2. Sample from system QA RBM and from system B at temperature $1/\beta_0$.
3. Compute expectation of $H(x)$ using the samples from QA and from B, respectively.
4. Update β using Eq. (56).
5. Repeat steps 2 to 4 until a convergence criterion is fulfilled..

Afterwards, $\beta_T \approx \beta_{QA}$. Therefore, rescaling the Hamiltonian by $1/\beta_T$ and then parsing the new Hamiltonian parameters onto the QA via Eqs. (49) will ensure that we are effectively sampling from H. Notice that in the previous method, $\langle H(x) \rangle_{QA}$ is independent of β, hence in the previous algorithm we need to generate samples only from system B every time we update β. This can be rather inconvenient, for instance, in the case where our interest is in having the QA mimic B, where the latter is a trained RBM. Changing the temperature of system B to match that of QA will affect the performance of the model. We might be tempted to invert the method and fit β_{QA}, but this is not a viable approach, since one does not have control over β_{QA} let alone a measurement of it. Instead, one can do the following: Replace the original Hamiltonian with that scaled by β, *i.e.*, $H(x) \to H(x)/\beta$, which leads to:

$$\beta_{t+1} = \beta_t - \eta \left(\langle H(x) \rangle_{QA^{(r)}} - \langle H(x) \rangle_{B(1)} \right) \tag{57}$$

where $QA^{(r)}$ correspond to rescaling $H(x)$ by $1/\beta_t$. In reaching the previous equation, we redefined $\frac{\eta}{\beta_t} \to \eta$ where η is fixed. The pseudo-algorithm to fit β using Eq. (57) is:

1. Fix learning rate η and initialize β_0. Parse the RBM parameters onto the Quantum Annealer via multiplying Eqs. (49) by $1/\beta_0$.
2. Sample from the QA RBM and from the B RBM at temperature 1.
3. Compute expectation of $H(x)/\beta_0$ using the samples from QA and from B, respectively.
4. Update β_1 using Eq. (57).
5. Repeat steps 2 to 4 until a convergence criterion is fulfilled.

The previous method is one of the common approaches used to estimate the β_{QA} parameter in QAs. However, it can be slow to converge which is why we propose a simple mapping with a stable fixed point at β_{QA}. We describe the method in full detail in the following.

F.4 DWave β_{QA} parameter estimation: Method 2

Once again, let us assume two RBMs which we denote as QA and B, both, described by the same Hamiltonian at different temperatures, *viz.*:

$$P_{QA}(x) = \frac{e^{-\beta_{QA} H(x)}}{Z(\beta_{QA})} \,, \tag{58}$$

$$P_B(x) = \frac{e^{-\beta H(x)}}{Z(\beta)} \,. \tag{59}$$

We denote as β_{QA} and β the inverse temperatures of system QA and B, respectively. Now, let us denote as S_{QA} and S_B as the entropy of QA and B, respectively, and assume $S_{QA} = S_B$, from which after some straightforward algebra:

$$\beta = \beta_{QA} \frac{\langle H \rangle_{QA}}{\langle H \rangle_{B(\beta)}} + \frac{\ln \frac{Z(\beta_{QA})}{Z(\beta)}}{\langle H \rangle_{B(\beta)}} \,. \tag{60}$$

We can further simplify the previous expression by introducing the variable $\Delta\beta = \beta_{QA} - \beta$:

$$\beta = \beta_{QA}\frac{\langle H\rangle_{QA}}{\langle H\rangle_{B(\beta)}} + \frac{\ln\langle e^{-\Delta\beta H}\rangle_{B(\beta)}}{\langle H\rangle_{B(\beta)}}\,. \tag{61}$$

Notice that the r.h.s. of Eq. (61) has a fixed point at $\beta = \beta_{QA}$. Here on we will only keep the first term in the r.h.s. and we will show that the fixed point is stable. In addition, same as we did when deriving the previous method, we replace $H(x) \to H(x)/\beta$. Since we do not have any control over β_{QA} nor we know the value *a priori*, we replace the prefactor in the first term of the r.h.s. with β since it does not affect the fixed point value and we further introduce a stability parameter $\delta(>0)$. After the previous considerations, we propose the following mapping:

$$\beta_{t+1} = f_\delta(\beta_t) \equiv \beta_t\left(\frac{\langle H\rangle_{QA^{(r)}}}{\langle H\rangle_{B(1)}}\right)^\delta \tag{62}$$

The function f_δ has a fixed point at $\beta = \beta_{QA}$. The stability condition close to the fixed point correspond to $|f'_\delta(\beta_{QA})| < 1$. The first derivative at the fixed point yields:

$$|f'_\delta(\beta_{QA})| = \begin{cases} |1 + \frac{\sigma^2_{QA}}{\langle H\rangle_{B(1)}}|,\ \delta = 1 \\ |1 + \delta\frac{\sigma^2_{QA}}{\langle H\rangle_{QA}}|,\ \delta \neq 1\,. \end{cases} \tag{63}$$

In the Methods section we have plotted Eq. (63) *vs* β for different values of δ. The values of β chosen for this plot correspond to where we typically find the fixed point. We call δ a stability parameter since we can tune it to stabilize the mapping per iteration.

A similar analysis can be done for the previous method. Specifically, the stability condition becomes:

$$|1 - \frac{\sigma^2_{QA}}{\beta_{QA}/\eta}| < 1\,. \tag{64}$$

From the previous it is easy to notice that the fixed point is unstable when the learning rate, η, such that $\eta > \beta_{QA}/\sigma^2_{QA}$ ($\beta_{QA}/\sigma^2_{QA} \sim 2\cdot 10^{-2}$).

G Incident energy conditioning

In the conditioned Calo4pQVAE framework, we condition the latent space RBM using the incident energy. We perform the condition as follows:

1. By applying a floor function on the incident energy in MeV, bin the incident energy, e, $e_{bin} = \text{floor}(e)$; the logarithm of the incident energy multiplied by 10, $e^{\ln}_{bin} = \text{floor}(10\cdot\ln e)$; and the square root of the incident energy multiplied by 10, $e^{\sqrt{}}_{bin} = \text{floor}(10\cdot\sqrt{e})$.
2. Convert to binary number the three previous binned numbers, $B_e = \text{binary}(e_{bin})$, $B_{\ln e} = \text{binary}(e^{\ln}_{bin})$, $B_{\sqrt{e}} = \text{binary}(e^{\sqrt{}}_{bin})$. We allocate 20 bits for each of these binary numbers.
3. Concatenate the three binary numbers, $B = \text{cat}(B_e, B_{\ln e}, B_{\sqrt{e}})$.
4. Use one partition to fit as many repetitions of the concatenated binary number B.
5. Set residual nodes to zero.

We fixed the number of nodes per partition to 512, hence the binary number B fits 8 times and the number of residual nodes is 32.

H Gaussian approximation to shower logits

In the main text we describe the data transformation used to train our model, where we first reduce the voxel energy per event by dividing it by the incident energy and we afterwards construct logits based on the reduced energy random variable. The number of particles in the electromagnetic shower follows approximately a Poisson distribution. Furthermore, via the saddle point approximation, for large number of particles in the shower the

multivariate Poisson distribution becomes a multivariate Gaussian distribution with the mean equal to the variance. Here we show that to zeroth approximation, the logits are Gaussian distributed.

Let us consider a Gaussian positive distributed random variable r with mean and variance Λ, *i.e.*,

$$f(r) = \frac{\mathcal{N}(r|\Lambda,\Lambda)}{\Omega}, \forall r > 0 \tag{65}$$

where Ω is a normalization constant. We define $u = \ln \frac{x}{1-x}$ with $x = r/R$ and $R \gg r$. To zeroth order approximation, $u \approx \ln r - \ln R$. To obtain the distribution of u we first introduce an auxiliary random variable $z = \ln r$ with distribution $g(z)$. By equating the cumulatives of r and z we obtain:

$$g(z) = e^z f(e^z)\,. \tag{66}$$

The distribution of u, $h(u)$, is simply the distribution of z shifted by $\ln R$, namely, $h(u) = g(u + \ln R)$:

$$h(u) = \frac{Re^u}{\Omega}\frac{1}{\sqrt{2\pi\Lambda}}e^{-\frac{(Re^u-\Lambda)^2}{2\Lambda}}\,. \tag{67}$$

The previous distribution is highly sensitive to u and the main contribution comes from $Re^u \approx \Lambda$. Hence, we can expand $\ln r$ around $\ln \Lambda$:

$$\ln r \approx \ln \Lambda + \frac{r-\Lambda}{\Lambda}, \tag{68}$$

which translates to $u \approx \ln \frac{\Lambda}{R} + \frac{r-\Lambda}{\Lambda}$. Notice that since $\frac{r-\Lambda}{\Lambda} \sim \mathcal{N}(0, \frac{1}{\Lambda})$, then:

$$u \sim \mathcal{N}(\ln \frac{\Lambda}{R}, \frac{1}{\Lambda})\,. \tag{69}$$

References

[1] Diederik P Kingma and Max Welling. Auto-encoding variational bayes. *arXiv preprint arXiv:1312.6114*, 2013.

[2] George EP Box and Mervin E Muller. A note on the generation of random normal deviates. *The annals of mathematical statistics*, 29(2):610–611, 1958.

[3] Chris J Maddison, Andriy Mnih, and Yee Whye Teh. The concrete distribution: A continuous relaxation of discrete random variables. *arXiv preprint arXiv:1611.00712*, 2016.

[4] Matej Balog, Nilesh Tripuraneni, Zoubin Ghahramani, and Adrian Weller. Lost relatives of the gumbel trick. In *International Conference on Machine Learning*, pages 371–379. PMLR, 2017.

[5] Amir H Khoshaman and Mohammad Amin. Gumbolt: Extending gumbel trick to boltzmann priors. *Advances in Neural Information Processing Systems*, 31, 2018.

[6] Aurélien Decelle, Cyril Furtlehner, and Beatriz Seoane. Equilibrium and non-equilibrium regimes in the learning of restricted boltzmann machines. *Advances in Neural Information Processing Systems*, 34:5345–5359, 2021.

[7] JJ Sakurai and Jim Napolitano. *Modern Quantum Mechanics*. Cambridge University Press Cambridge, 2017.
